# Biochemical and Flavour Profile of Lingzhi (*Ganoderma lucidum*) Mycelium Fermented by *Lactiplantibacillus plantarum*: A Dual-Modelling Approach to Umami Synergy and Antioxidant Capacity

**DOI:** 10.3390/antiox15091196

**Published:** 2026-09-18

**Authors:** Yutong Gu, Zhiyu Zhao, Rushan Lakshitha, Tony Chen, Kevin Kantono, Wan Abd Al Qadr Imad Wan-Mohtar, Siva Raseetha, Nazimah Hamid

**Affiliations:** 1 School of Science, Faculty of Health and Environment Sciences, Auckland University of Technology, Auckland 1010, New Zealandkkantono@aut.ac.nz (K.K.); 2 Functional Omics and Bioprocess Development Laboratory, Institute of Biological Sciences, Faculty of Science, Universiti Malaya, Kuala Lumpur 50603, Malaysia; qadyr@um.edu.my; 3 Faculty of Applied Sciences, Universiti Teknologi MARA, Shah Alam 40450, Selangor, Malaysia; raseetha@uitm.edu.my

**Keywords:** Lingzhi mushroom mycelium, fermentation time, antioxidant activity, bioactive compounds, *Lactiplantibacillus plantarum*, PLSR, umami synergy, taste-active metabolites

## Abstract

The broader application of Lingzhi (*Ganoderma lucidum*) in functional foods is fundamentally restricted by its intense, triterpenoid-driven bitterness. This study investigated the biochemical changes in Lingzhi mushroom mycelium (LMM) over a 10-day controlled fermentation with *Lactiplantibacillus plantarum*. To evaluate bioactivity enhancement, a dual predictive modelling strategy (Partial Least Squares Regression [PLSR] and elastic-net) was applied to the non-volatile metabolite matrix, identifying model-supported candidate contributors to TPC and FRAP antioxidant responses. Concurrently, fermentation significantly transformed the matrix, yielding an estimated 24-fold increase in synergistic umami intensity, with Equivalent Umami Concentration (EUC) increasing from 0.27 to 6.49 g MSG/100 g fermented LMM by Day 10. This increase was primarily driven by the accumulation of glutamic acid (Taste Activity Value [TAV] = 1.27) and its potent synergy with 5′-GMP (TAV = 2.82). Furthermore, Principal Component Analysis (PCA) of GC-MS volatile data revealed a distinct temporal transition from lipid-derived aldehydes to microbially generated ‘mushroom’ alcohols and mid-fermentation esters, suggesting enhanced aromatic complexity. These findings indicate that targeted *L. plantarum* fermentation can convert raw LMM into a bioactive ingredient with enhanced umami and antioxidant capacity suggesting potential for improved palatability that warrants sensory confirmation.

## 1. Introduction

In recent years, global mushroom consumption has significantly increased due to mushrooms’ recognised nutritional and medicinal value [1]. *Ganoderma lucidum*, known as Lingzhi, is a well-known medicinal mushroom traditionally used in China and esteemed for its health-promoting properties. Its significance lies in a rich profile of bioactive compounds found in both its fruiting body and mycelium, including polysaccharides and triterpenoids, which are responsible for benefits such as immunomodulatory and antioxidant activities [2]. However, the broader application of Lingzhi in the functional food sector is severely limited by its inherent, astringent bitterness that is driven primarily by these triterpenoids, which limits consumer acceptability.

From a production and compositional standpoint, Lingzhi mushroom mycelium (LMM) offers a favourable solution to both agricultural and nutritional challenges. While traditional mushroom fruiting bodies are prone to decay and require a lengthy growth period of approximately six months, mushroom mycelium offers greater structural resilience and a significantly shorter growth cycle of about three weeks, a process accelerated by technological advancements like submerged fermentation [3]. Mushroom mycelium shares many beneficial compounds found in fruiting bodies, exhibiting strong antioxidant, anti-inflammatory, and anticarcinogenic properties, which positions it as a valuable nutritional supplement [2]. Studies indicate that mycelium can serve as a rich source of bioactives with stronger antioxidant effects compared to mature fruiting bodies [4]. Mycelium extracts have also been shown to have a higher concentration of vitamin D, presenting an opportunity for fortified health foods [5]. This efficiency, combined with its high nutritional value, makes mycelium utilisation a promising step towards promoting sustainability and a scalable functional food base [6].

Microbial fermentation serves as a critical bioprocessing tool to transform raw LMM into a palatable food ingredient. For *G. lucidum*, the duration of fermentation can strongly influence the yield of its bioactive compounds and shape the sensory characteristics of the final product. Recent studies highlight that fermentation with lactic acid bacteria, particularly *Lactiplantibacillus plantarum*, is an effective strategy for modifying complex food matrices, as it has been reported to reduce undesirable bitter compounds, and promote the accumulation of umami-related free amino acids and nucleotides [7,8]. While the dynamic nature of antioxidant activity and the general formation of flavour compounds are recognised, how microbial metabolism may mitigate Lingzhi’s specific triterpenoid bitterness while building savoury characteristics remains poorly understood. Furthermore, recent studies emphasise that the dynamic interaction between non-volatile taste-active compounds cannot be understood through simple chemical quantification alone. Evaluating complex taste mechanisms such as the synergy between amino acids and 5′-nucleotides, or sub-threshold taste-taste interactions, requires metrics like Equivalent Umami Concentration (EUC) and Taste Activity Values (TAVs), which provide model-based estimates of perceived taste intensity [9]. Therefore, optimising fermentation time is essential to maximise the desired antioxidant properties while ensuring a palatable flavour profile. 

This study aimed to investigate the biochemical changes in LMM during a 10-day controlled fermentation with *L. plantarum*. Recognising the complex nature of mushroom mycelium flavour, the objectives were to: (1) use a dual multivariate modelling strategy combining Partial Least Squares Regression (PLSR) and elastic-net regularised regression to isolate the ‘consensus candidate contributors’ associated with peak antioxidant capacity; (2) quantitatively model the synergistic Equivalent Umami Concentration (EUC) and individual Taste Activity Values (TAVs) to evaluate umami enhancement and the potential for sub-threshold masking of bitter notes; and (3) track the transition of the volatile aroma profile. Ultimately, this study sought to establish how fermentation time can transform LMM into a high-value ingredient that combines enhanced bioactivity and umami intensity, potentially supporting improved palatability as a natural flavour enhancer.

## 2. Materials and Methods

### 2.1. Materials

Lingzhi mushroom mycelium (LMM) powder was sourced from iHerb, LLC (Irvine, CA, USA). The *Lactiplantibacillus plantarum* strain was obtained from the culture collection of the School of Science, Auckland University of Technology (AUT, Auckland, New Zealand), and no commercial strain identifier is available for this strain. BD GasPak™ EZ Anaerobe Container System sachets, BD BBL™ GasPak™ anaerobic jars, and BD Difco™ *Lactobacilli* MRS (De Man, Rogosa and Sharpe) broth were all supplied by Thermo Fisher Scientific (Auckland, New Zealand). *Lactobacillus* MRS agar was obtained from Fort Richard Laboratories (Auckland, New Zealand).

### 2.2. Fermentation of LMM with L. plantarum

To prepare the inoculum, 100 mL of BD Difco™ *Lactobacilli* MRS broth was first inoculated with 1 mL of *Lactiplantibacillus plantarum* reserve culture (stored in 20% glycerol at −80 °C). The inoculated broth was transferred to a BD BBL™ anaerobic jar containing BD GasPak™ EZ Anaerobe Container System sachets and incubated at 35 °C for 24 h under anaerobic conditions. Following incubation, culture purity and cell morphology were verified using Gram staining. A working starter culture was then prepared by subculturing 1 mL of this pure stock with an approximate bacterial suspension of 9.0 × 10^8^ CFU/mL (equivalent to a 3.0 McFarland standard) into 100 mL of fresh MRS broth, which was incubated anaerobically at 35 °C, as described in a previously reported paper [10]. To initiate the solid-state fermentation, 9 g of LMM powder, 9 mL of sterile peptone water, and 0.9 mL of the prepared *L. plantarum* starter culture were aseptically combined in a sterile plate and mixed thoroughly to ensure uniform distribution.

To monitor bacterial growth kinetics, a sample of the fermenting LMM mixture (1 g) was aseptically collected on Day 0. The sample was suspended in 9 mL of sterile 0.1% peptone water and homogenised thoroughly by vortexing. Ten-fold serial dilutions (10^−1^ to 10^−7^) were prepared in 0.1% peptone water, and 0.1 mL aliquots of the appropriate dilutions were spread-plated onto MRS agar. The plates were incubated under anaerobic conditions at 35 °C. This sampling and enumeration procedure was repeated daily throughout the 10-day fermentation period (Days 0 to 10). Finally, viable cell counts were recorded as colony-forming units per gram (CFU/g) and plotted against fermentation time to establish the growth curve. The detection limit of this enumeration method was 10^2^ CFU/g, corresponding to plating 0.1 mL of the 10^−1^ dilution.

#### 2.2.1. Solid-State Fermentation and Sampling Strategy

To account for biological variance, three independent replicates were prepared for each daily time point (Days 0 to 10), resulting in a total cohort of 33 individual fermentation plates. All 33 plates were immediately transferred into BD BBL™ anaerobic jars. To establish and maintain strict anaerobic conditions, three BD GasPak™ EZ Anaerobe Container System sachets were added to the vessels. The jars were then placed in an incubator at a constant temperature of 35 °C.

Sampling was conducted every 24 h. At each daily time point, three plates (representing one biological triplicate) were aseptically removed from the incubator. To ensure the integrity of the anaerobic environment for the remaining plates, the three BD GasPak™ sachets were completely replaced following each daily opening of the jars. Upon collection, the daily LMM samples were immediately sealed in sterile bags and transferred to a −80 °C freezer to halt microbial metabolism and preserve the metabolite profile prior to downstream biochemical and flavour modelling analyses.

#### 2.2.2. Metabolite Extraction

To extract the target metabolites, a 0.1 g aliquot of the fermented LMM sample was weighed into a centrifuge tube and suspended in 10 mL of absolute methanol (Merck, Darmstadt, Germany). The mixture was homogenised using a T-25 Ultra-Turrax homogeniser (IKA Janke & Kunkel, Staufen, Germany) at 12,000 rpm for 90 s, followed by a 1 min resting period to ensure complete solvent penetration. Subsequently, the homogenate was centrifuged at 1500 rpm for 15 min at 4 °C using an Eppendorf 5810R refrigerated centrifuge (Eppendorf, Hamburg, Germany). The resulting upper supernatant was carefully decanted and transferred into a 10 mL volumetric flask. The final methanolic extract was then sealed and stored at −20 °C until required for downstream analysis. This standard extraction protocol was applied to all 33 biological replicates collected across the 10-day fermentation period.

### 2.3. LC-MS Analysis of Organic Acids

The quantification of organic acids in the fermented LMM extracts was modified from Zhao et al. [11] and LC-MS analyses were performed using an Agilent 1260 Infinity Quaternary LC system connected to an Agilent 6420 Triple Quadrupole Mass Spectrometer (Agilent Technologies, Santa Clara, CA, USA). Chromatographic separation was achieved on an Agilent Poroshell 120 Aq-C18 column (2.1 × 150 mm, 2.7 µm). The mobile phase consisted of Milli-Q water containing 0.1% (*v*/*v*) formic acid (eluent A) and methanol (eluent B), delivered at a constant flow rate of 0.2 mL/min. The elution gradient was programmed as follows: the initial composition was set at 97% A; from 3 to 5 min, eluent B was linearly increased to 50%; and from 10 to 11 min, eluent B was reduced back to 3% to re-equilibrate the column. The total run time was 25 min per sample, with an injection volume of 3 µL.

Mass spectrometric detection was carried out using an electrospray ionisation (ESI) source operating in negative ion mode. The MS source parameters were optimised as follows: capillary voltage, 4 kV; drying gas temperature, 300 °C; drying gas flow rate, 10 L/min; and nebuliser pressure, 30 psi. Quantitative analysis was conducted using Multiple Reaction Monitoring (MRM). All instrumental analyses were carried out in biological triplicate.

### 2.4. LC-MS Analysis of Free Amino Acids

Following the methodology described by Dong et al. [12] with minor modifications, chromatographic separation was performed using an Agilent Poroshell EC-C18 column (2.1 × 150 mm, 2.7 µm) maintained at 40 °C. The mobile phase consisted of water containing 0.1% (*v*/*v*) formic acid (eluent A) and acetonitrile containing 0.1% (*v*/*v*) formic acid (eluent B), delivered at a constant flow rate of 0.3 mL/min. The elution gradient was programmed as follows: 0–1 min, 5% B; 1–5 min, linear increase to 10% B; 5–10 min, increase to 15% B; 10–15 min, increase to 45% B; 15–17 min, increase to 80% B; 17–19 min, isocratic hold at 80% B; and 19–20 min, reduction back to 5% B. The system was then allowed to re-equilibrate at 5% B for 9 min, resulting in a total run time of 29 min per sample.

Mass spectrometric detection parameters were optimised as follows: capillary voltage, 4 kV; drying gas temperature, 300 °C; drying gas flow rate, 10 L/min; and nebuliser pressure, 30 psi. Analysis was conducted in positive ESI mode.

### 2.5. LC-MS Analysis of 5′-Nucleotide Assays

The quantification of five 5′-nucleotides was carried out by LC-MS/MS. The method was adapted from Viñas et al. [13] and Dong et al. [12] with modifications. The targeted analytes were cytidine 5′-monophosphate (5′-CMP), uridine 5′-monophosphate (5′-UMP), adenosine 5′-monophosphate (5′-AMP), guanosine 5′-monophosphate (5′-GMP), and inosine 5′-monophosphate (5′-IMP), using an Agilent 1260 Infinity Quaternary LC system coupled to an Agilent 6420 Triple Quadrupole Mass Spectrometer equipped with an electrospray ionisation (ESI) source (Agilent Technologies, Santa Clara, CA, USA). Chromatographic separation was achieved on an Agilent Poroshell 120 Aq-C18 column (2.1 × 150 mm, 2.7 µm). The mobile phase consisted of water containing 0.1% (*v*/*v*) formic acid (eluent A) and acetonitrile containing 0.1% (*v*/*v*) formic acid (eluent B), delivered at a constant flow rate of 0.2 mL/min. The elution gradient was programmed as follows: 0–2 min, 2% B; 2–4 min, linear increase to 50% B; 4–6 min, increase to 80% B; 6–9 min, isocratic hold at 80% B; and 9–10 min, reduction back to 2% B. The system was then allowed to re-equilibrate at 2% B for 7 min, resulting in a total run time of 17 min per sample.

Mass spectrometric detection was executed in negative ion mode. The MS source parameters were optimised as follows: capillary voltage, 4 kV; drying gas temperature, 300 °C; drying gas flow rate, 10 L/min; and nebuliser pressure, 30 psi.

### 2.6. LC-MS Analysis of Reducing Sugars

LC-MS analysis of reducing sugars was performed following pre-column derivatisation with 1-phenyl-3-methyl-5-pyrazolone (PMP), modified from the method described by Ortiz-Tena et al. [14] with minor modifications. Briefly, the extracted samples were derivatized with PMP in the presence of sodium hydroxide at 70 °C for 30 min, followed by neutralisation with hydrochloric acid and extraction with chloroform to remove excess PMP reagent. LC-MS analysis was conducted using an Agilent 1260 Infinity Quaternary LC system coupled to an Agilent 6420 Triple Quadrupole Mass Spectrometer equipped with an electrospray ionisation (ESI) source (Agilent Technologies, Santa Clara, CA, USA). Chromatographic separation was achieved on a Phenomenex Kinetex EVO C18 column (2.1 × 150 mm, 1.7 µm). The mobile phase consisted of water containing 0.1% (*v*/*v*) formic acid (eluent A) and acetonitrile containing 0.1% (*v*/*v*) formic acid (eluent B), delivered at a constant flow rate of 0.25 mL/min with an injection volume of 2 µL. The elution gradient was programmed as follows: 0–13 min, isocratic hold at 17% B; 13–14 min, linear increase to 80% B; and 14–16 min, reduction back to 17% B. The system was then allowed to re-equilibrate at 17% B for 6 min, resulting in a total run time of 22 min per sample.

Mass spectrometric detection was executed in positive ion mode. The MS source parameters were optimised as follows: capillary voltage, 4 kV; drying gas temperature, 300 °C; drying gas flow rate, 10 L/min; and nebuliser pressure, 30 psi. The LC-MS/MS methods were validated following the protocol described in Zhao et al. [11] on the same instrument platform (Agilent 1260 Infinity Quaternary LC-6420 triple quadrupole MS) and by the same analyst. The calibration ranges were 0.156–20 mg/L (R^2^ ≥ 0.9934) for the organic acids and 7.8-1000 µmol/L (R^2^ = 0.9988–0.9995, weighted fits) for the 5′-nucleotides, reflecting the mass-based and molar-based preparation of the respective standard stock solutions. Limits of detection (LODs) and quantification (LOQs) were calculated as LOD = 3.3 × S_residuals_/slope and LOQ = 10 × S_residuals_/slope, where S_residuals_ is the standard deviation of the residuals of the calibration curve. For the organic acids, the residuals of a linear fit were systematically curved across the calibration range (from −2.0% at the highest standard to −44.2% at the lowest concentration), indicating model mis-specification; the calibration curves were therefore re-fitted with second-order polynomials (R^2^ = 0.99971–0.99998), yielding LOD/LOQ of 0.415/1.258 mg/L (malic acid; after exclusion of the 2.5 mg/L standard, whose back-calculated accuracy deviated by 20% under the quadratic fit), 0.094/0.285 mg/L (lactic acid) and 0.292/0.884 mg/L (succinic acid). For the five 5′-nucleotides, 1/x-weighted least-squares regression was applied over the eight-level calibration range (7.8–1000 µmol/L), because the response variance increased with concentration. Calibration points whose back-calculated accuracy fell outside 80–120% were excluded from the regression (CMP: 31.25 and 7.81 µmol/L, at 128.7% and 153.6%; AMP: 15.63 and 7.81 µmol/L, at 148.4% and 530.1%; IMP: 7.81 µmol/L, at 200.4%). A re-injection of the 62.5 µmol/L mixed standard at the end of the analytical sequence served as a continuing calibration verification, with accuracies of 78.2–90.3% across the five analytes, confirming negligible drift over the run. The resulting LOD/LOQ values were 1.8/5.3, 2.0/6.0, 1.3/4.0, 1.7/5.0 and 1.7/5.1 µmol/L for CMP, UMP, AMP, GMP and IMP, respectively. The free amino acid and reducing sugar methods were validated as reported in the literature [15,16].

### 2.7. Total Phenolic Content and Antioxidant Capacity

#### 2.7.1. Determination of Total Phenolic Content (TPC)

The total phenolic content of the fermented LMM extracts was determined using the Folin–Ciocalteu assay according to Zhao et al. [17]. To construct the calibration curve, a 1.0 mg/mL stock solution was prepared by dissolving 0.1 g of gallic acid (Sigma-Aldrich, Auckland, New Zealand) in deionised water within a 100 mL volumetric flask. Working standard solutions with concentrations of 2.5, 5, 10, 20, 40, and 80 mg/L were subsequently prepared via dilution with deionised water. All TPC, CUPRAC and FRAP values are expressed per gram of fermented matrix on a wet basis (g of fermented LMM), consistent with the reporting basis described in Section 2.10.

For the assay, 1 mL of either the methanolic LMM extract (obtained in Section 2.2.2) or the standard solution was combined with 0.5 mL of Folin–Ciocalteu reagent (Sigma-Aldrich, New Zealand) in a glass vial. The mixture was allowed to react for 5 min at room temperature. Subsequently, 1.5 mL of a 20% (*w*/*v*) Na_2_CO_3_ solution (Sigma-Aldrich, New Zealand) was added. The solution was mixed thoroughly and incubated in the dark at room temperature for 2 h. Absorbance was then measured at 765 nm using an Ultrospec 2100 Pro spectrophotometer (Amersham Pharmacia Biotech, Buckinghamshire, UK). The TPC was calculated using the standard curve and expressed as milligrams of gallic acid equivalents per gram of fermented matrix (wet basis) (mg GAE/g fermented matrix (wet basis)). All determinations were performed in biological triplicate.

#### 2.7.2. Determination of Cupric Reducing Antioxidant Capacity (CUPRAC)

The CUPRAC assay was employed to determine the antioxidant activity of the fermented LMM extracts as described by Zhao et al. [10]. To construct the calibration curve, a 1000 mg/L stock solution was prepared by dissolving 0.1 g of ascorbic acid (Sigma-Aldrich, New Zealand) in deionised water within a 100 mL volumetric flask. Working standard solutions with concentrations of 2.5, 5, 10, 20, 40, and 80 mg/L were subsequently prepared via dilution with deionised water.

For the assay, 1 mL of either the methanolic LMM extract (obtained in Section 2.2.2) or deionised water (as a blank) was combined with 1 mL of 0.01 M copper(II) chloride (CuCl_2_), 1 mL of 1 M ammonium acetate buffer (NH_4_Ac, pH 7.0), 1 mL of 0.075 M neocuproine, and 0.1 mL of deionised water in a glass vial, yielding a final reaction volume of 4.1 mL. All chemical reagents were sourced from Sigma-Aldrich (Auckland, New Zealand). The reaction mixture was vortexed thoroughly and incubated for 5 min at room temperature. Absorbance was then measured at 450 nm using an Ultrospec 2100 Pro spectrophotometer (Amersham Pharmacia Biotech, Buckinghamshire, UK). The antioxidant capacity was calculated using the standard curve and expressed as milligrams of ascorbic acid equivalents per gram of fermented matrix (wet basis) (mg AAE/g fermented matrix (wet basis)). All determinations were performed in biological triplicate.

#### 2.7.3. Determination of Ferric Reducing Antioxidant Power (FRAP)

The FRAP assay was used to evaluate the antioxidant capacity of the fermented LMM extracts, following the procedure described by Ioannou et al. [18]. The ascorbic acid standard solutions, used to construct the calibration curve, were prepared exactly as described for the CUPRAC assay (Section 2.7.2). To prepare the working FRAP reagent, 300 mM acetate buffer, 10 mM 2,4,6-tris(2-pyridyl)-s-triazine (TPTZ), and 20 mM iron(III) chloride (FeCl_3_) were mixed in a 10:1:1 (*v*/*v*/*v*) ratio. All chemical reagents were sourced from Sigma-Aldrich (Auckland, New Zealand). This reagent mixture was pre-warmed in a 36 °C water bath prior to use.

For the assay, 0.1 mL of the methanolic sample extract (or deionised water for the blank) was combined with 0.9 mL of deionised water and 2 mL of the pre-warmed FRAP reagent in a spectrophotometric cuvette (Interlab, Auckland, New Zealand). The solution was mixed well and allowed to react for 4 min at room temperature. Absorbance was subsequently measured at 593 nm using an Ultrospec 2100 Pro spectrophotometer (Amersham Pharmacia Biotech, UK). The FRAP values were calculated using the standard curve and expressed as milligrams of ascorbic acid equivalents per gram of fermented matrix (wet basis) (mg AAE/g fermented matrix (wet basis)). All determinations were performed in biological triplicate.

### 2.8. SPME-GC-MS Analysis of Volatile Flavour Compounds

The profiling of volatile compounds in the fermented LMM was conducted using the Agilent 7890A Gas Chromatograph (GC) equipped with the Gerstel Multi-Purpose Autosampler and a 5977A mass spectrometer detector (MSD) (Agilent Technologies, Santa Clara, CA, USA), following the methodology described by Ma et al. [19] with minor modifications. To ensure comprehensive volatile extraction and resolution, chromatographic separation was performed using two distinct Agilent capillary columns: a polar DB-FATWAX UI (30 m × 0.25 mm, 0.25 µm film thickness) and a slightly non-polar DB-5MS UI (30 m × 0.25 mm, 0.25 µm film thickness). Briefly, 1 g of each sample was sealed in a 10 mL amber vial and extracted using a CAR/DVB/Carboxen fibre (Supelco, Bellefonte, PA, USA, PN: 57329-U) after incubation at 50 °C for 30 min, followed by 15 min of extraction; desorption was performed in the GC inlet at 250 °C in splitless mode for 2 min.

For both column configurations, the inlet temperature was maintained at 250 °C, and samples were injected in splitless mode using helium as the carrier gas at a constant flow rate of 1.1 mL/min. The oven temperature program was initiated at 40 °C (held for 2 min), ramped at 8 °C/min to 240 °C, and held isothermally for 3 min, resulting in a total run time of 30 min. 

Mass spectrometric detection was performed in electron impact (EI) mode at 70 eV. The transfer line, ion source, and quadrupole temperatures were maintained at 250 °C, 250 °C, and 150 °C, respectively. Data acquisition was conducted in full-scan mode over a mass range of 38–450 Da, following a 1 min solvent delay. Volatile compounds were identified by matching their mass fragmentation patterns with the NIST14 mass spectral library and comparing their calculated Kovats retention indices (RIs). The RI values were determined using a homologous series of n-alkanes (C8–C30) analysed under identical chromatographic conditions. As identification was based on library matching and retention indices without confirmation by authentic standards, all reported volatile compounds were considered tentatively identified. The relative abundance of each identified volatile compound was semi-quantitatively expressed as the ratio of its specific peak area to the total peak area of all identified volatile compounds. Volatile compounds were quantified as relative abundances, expressed as the percentage of the total peak area of all identified compounds in each sample, as no internal standard was used for quantification.

### 2.9. Statistical Analysis

Statistical analyses were performed using RStudio (version 2025.09.1, Posit, PBC, Boston, MA, USA) and ANOVA was performed using XLSTAT 2024 (Lumivero, Denver, CO, USA). Post hoc comparisons were conducted using Fisher’s Least Significant Difference (LSD) test following a significant F-test, with differences considered significant at *p* < 0.05. Results are expressed as mean ± standard deviation (SD) based on triplicate measurements per batch. 

#### 2.9.1. Partial Least Squares Regression (PLSR)

Multivariate integration of the non-volatile matrix and antioxidant responses was performed using a PLSR workflow. The dataset comprised 33 observations (three batches per day from Day 0 to Day 10). The X matrix included all 34 quantified non-volatile compounds, namely 5′-nucleotides, free amino acids, organic acids, and reducing sugars, while the Y matrix comprised total phenolic content (TPC), ferric reducing antioxidant power (FRAP), and cupric reducing antioxidant capacity (CUPRAC). Compound values were converted to numeric format, standardised names were used for plotting, and the X variables were transformed using log(1 + x) before autoscaling; the Y variables were also autoscaled. A two-component PLSR model was fitted to the X and Y matrices for the main descriptive visualisation; an additional four-component PLSR model was examined to generate supplementary PLS3–PLS4 projections and assess whether further antioxidant-response structure was present beyond the first two components. For the score plot and correlation biplot, the three replicate batches within each day were averaged to generate day centroids for clearer trajectory visualisation. Variable importance in projection (VIP) scores were3.1 calculated for TPC, FRAP, and CUPRAC models.

#### 2.9.2. Elastic-Net Regression

As a complementary sparse modelling approach, assay-specific elastic-net regression was applied to the integrated non-volatile dataset to identify a reduced subset of compounds associated with total phenolic content (TPC), ferric reducing antioxidant power (FRAP), and cupric reducing antioxidant capacity (CUPRAC). The dataset comprised 33 observations (three batches per fermentation day from Day 0 to Day 10). The predictor matrix (X) contained all 34 quantified non-volatile compounds, including 5′-nucleotides, free amino acids, organic acids, and reducing sugars, whereas the response matrix (Y) comprised TPC, FRAP, and CUPRAC. Compound values were converted to numeric format, compound names were standardised for plotting, and the X variables were transformed using log(1 + x) before autoscaling. Because elastic-net was used here primarily as an interpretation-focused sparse modelling approach rather than as a fully tuned predictive optimisation exercise, a fixed penalty setting was applied consistently across assays (α = 0.05, l1_ratio = 0.90; max_iter = 500,000). For the interpretation-focused full-data models, each response was standardised separately before fitting. Standardised coefficient bar plots were used to summarise the retained variables and their directions of association, and coefficient-path plots were generated across a predefined α path (log10 α from approximately −2.2 to 0.3 at fixed l1_ratio = 0.90) to visualise coefficient shrinkage and sign persistence. A model-derived day-wise contribution heatmap was calculated as the product of each full-model coefficient and the corresponding standardised compound value, averaged within fermentation day and arranged as D0_TPC, D0_FRAP, D0_CUPRAC through to D10_TPC, D10_FRAP, and D10_CUPRAC. Internal validation was performed separately from the descriptive full-data figures using day-averaged data (*n* = 11 fermentation days) as the effective independent unit. Leave-one-day-out cross-validation was applied for each assay-specific model, with scaling fitted within each training fold and then applied to the held-out day. Predictive performance was summarised using Q^2^, Pearson correlation between observed and cross-validated predicted values [r(obs,pred)], and RMSECV. To assess overfitting, day-level permutation testing was performed by randomly permuting the response values across days 100 times and recalculating Q^2^ under the same validation framework. Selection stability was additionally quantified as the frequency with which individual compounds were retained across cross-validation folds.

### 2.10. Calculation of Taste Impact Metrics (EUC and TAV)

To evaluate the theoretical sensory contribution of the quantified metabolites, two metrics were calculated: the Equivalent Umami Concentration (EUC) and the Taste Activity Value (TAV). The synergistic umami intensity of the LMM was calculated using the equation established by Yamaguchi et al. [20] (pp. 846–849):

$$Y=\sum a_{i}b_{i}+1218\left(\sum a_{i}b_{i}\right)\left(\sum a_{j}b_{j}\right)$$

where Y is the EUC value (g MSG equivalent/100 g); a_i_ is the concentration (g/100 g) of umami amino acids (Asp and Glu); b_i_ is the relative umami intensity of each amino acid (Glu = 1, Asp = 0.077); a_j_ is the concentration (g/100 g) of umami 5′-nucleotides (5′-GMP and 5′-IMP); b_j_ is the relative umami intensity of each nucleotide (5′-GMP = 2.3, 5′-IMP = 1); and 1218 is the synergy constant.

Prior to calculation, the raw LC-MS concentrations (µmol/L of extract) were mathematically converted to mass fractions (g/100 g fermented LMM, wet basis). This was achieved by converting the molar concentration to grams using the specific molar mass of each respective compound, and then adjusting for the extraction dilution factor (10 mL of solvent per 0.1 g of fermented sample) to determine the final mass per 100 g of fermented LMM (wet basis). As samples were extracted as collected, without a prior drying step, the values are reported as per gram of fermented matrix (wet basis) rather than per gram of dry matter. The fermentation substrate was prepared by rehydrating dry LMM powder with peptone water at a 1:1 (*w*/*w*) ratio, corresponding to an estimated Day 0 moisture content of ≈50% (wet basis). Day 0 moisture and pH were not determined analytically.

The TAV was used to assess the individual contribution of each taste-active compound to the overall flavour profile. For each compound, the TAV was calculated as the ratio of its mean concentration (three biological replicates) to its taste threshold. To ensure dimensional consistency for this ratio, the raw LC-MS extract concentrations (µmol/L) were first converted to mg/100 g of fermented LMM (wet basis) utilising compound-specific molar masses and the 1:100 extraction dilution ratio. The converted concentrations (mg/100 g) were then divided by their respective human taste thresholds (mg/100 g) as reported in the literature [21]. 

Compounds with a calculated TAV > 1 were considered to contribute significantly and directly to the perceived taste profile of the fermented mycelium, while compounds with a TAV < 1 were evaluated for potential sub-threshold synergistic or cross-modal masking effects.

## 3. Results and Discussion

### 3.1. Microbiological Analysis

**Figure 1:**
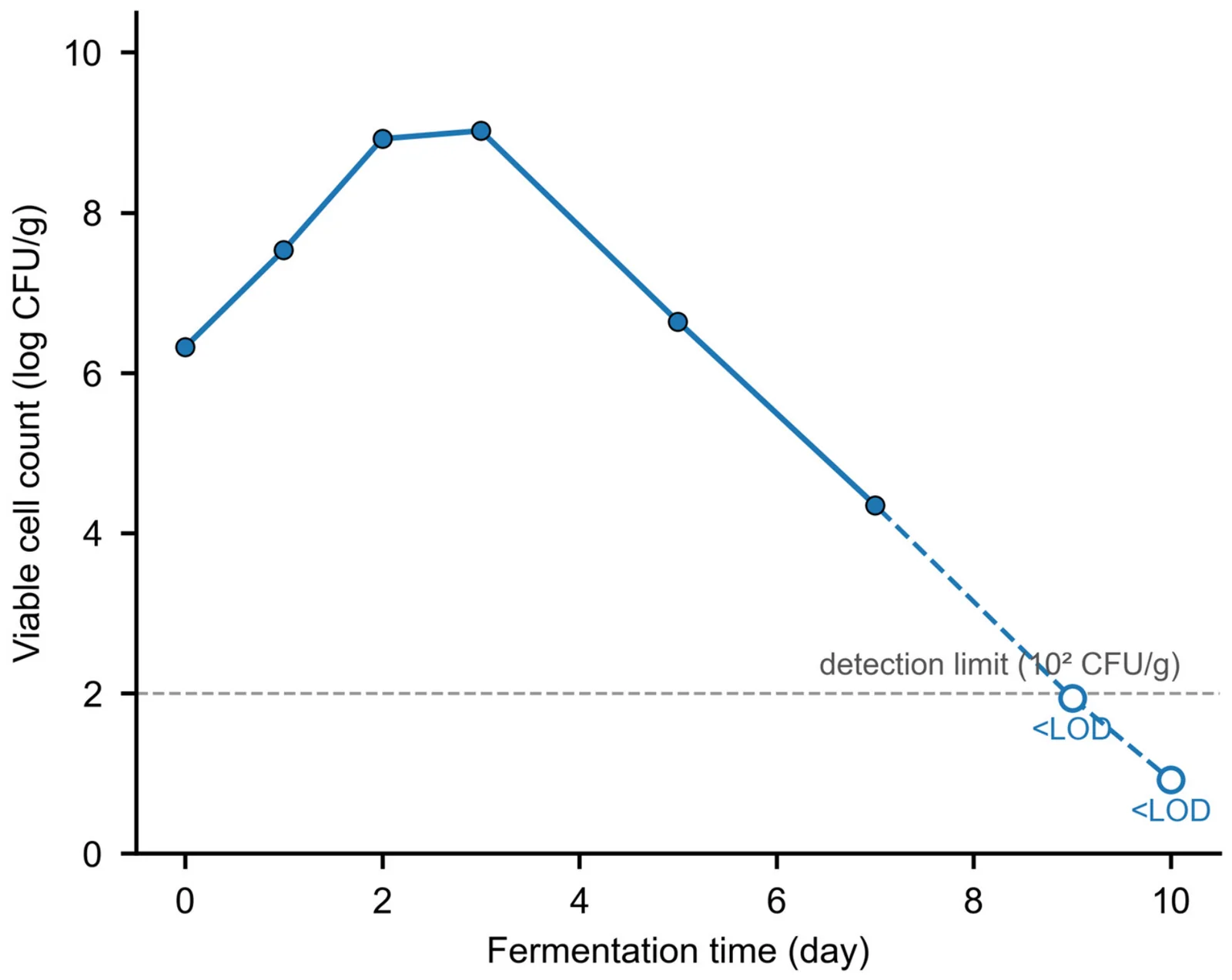
The relationship between fermentation time and colony number in LMM powder. Filled circles joined by a solid line show viable counts above the detection limit. Open circles joined by a dashed line show counts on Days 9 and 10, which fell below the detection limit of 10^2^ CFU/g and are therefore not quantifiable. The horizontal dashed line marks the detection limit.

The inoculated LMM was fermented from Day 0 to Day 10, for a total of eleven days of fermentation. The number of colonies on the MRS plate on each day was recorded and plotted as a curve with the number of days of fermentation as the horizontal coordinate and log(CFU) as the vertical coordinate (Figure 1).

The number of microorganisms in the fermentation substrate started to increase gradually from Day 0, peaked on Day 3, and then decreased gradually. This suggests that microorganisms are present in LMM and that co-inoculation with *L. plantarum* promoted their growth. The growth curve reflects the total viable microbiota (indigenous and inoculated) of the fermentation matrix. Some mutual interactions occurring between these microorganisms may also account for their favourable growth [22], whereas microbial population growth requires the consumption of nutrients, which is accelerated as the number of microorganisms increases. As the nutrients decrease, the microorganisms are unable to obtain the required nutrients to survive and hence the microbial population gradually decreases. On Days 9 and 10, viable counts fell below the enumeration detection limit (10^2^ CFU/g) and are therefore reported as below the limit of detection.

### 3.2. Effect of Fermentation on Organic Acid Content

The fermentation process significantly modulated the organic acid profile of the LMM (Table S1, Supplementary Materials). While malic acid levels declined, reaching their lowest point on Day 6, there was a marked accumulation of lactic and succinic acids, peaking on Day 9. This metabolic shift is consistent with the growth of the lactic acid bacterial population and provides a foundation for the observed changes in both flavour and bioactivity.

### 3.3. Effect of Fermentation on Free Amino Acid Content

A total of 22 free amino acids and ethanolamine (23 compounds in total) were quantified, with the majority showing a significant, time-dependent increase throughout the 10-day period (*p* < 0.05; Table S2, Supplementary Materials). Notably, the pool of umami and sweet-tasting amino acids, including glutamic acid and alanine, expanded substantially. These compounds serve as critical nitrogenous substrates for the subsequent umami synergy and antioxidant capacity models. 

### 3.4. Effect of Fermentation on 5′-Nucleotide Content

The 5′-nucleotide profile underwent significant changes, characterised by steady increases in 5′-CMP and 5′-GMP (Table S3, Supplementary Materials). Although 5′-IMP remained below the limit of detection (1.7 µmol/L) throughout, the enrichment of the 5′-CMP and 5′-GMP pool is a primary contributor to the ‘meaty’ or ‘brothy’ notes developed during fermentation. 

### 3.5. Effect of Fermentation on Reducing Sugar Content

Fermentation differentially influenced individual reducing sugars, with glucose exhibiting an initial surge followed by gradual consumption as a primary microbial carbon source (Table S4, Supplementary Materials). In contrast, arabinose showed a sustained accumulation, suggesting it is a non-preferential metabolic by-product in this specific fermentation matrix. 

### 3.6. Effect of Fermentation on Total Phenolic Content (TPC) and Antioxidant Properties

The antioxidant capacity of the LMM was significantly increased, with TPC and ferric reducing antioxidant power (FRAP) following clear upward trajectories (Table S5, Supplementary Materials). While CUPRAC exhibited a more complex, non-linear fluctuation, the overall increase in reducing power confirms the functional transformation of the mycelial matrix.

### 3.7. Partial Least Squares Regression (PLSR) and VIP Analyses of the Integrated Non-Volatile Matrix and Antioxidant Responses

To identify the compounds most strongly associated with the enhanced antioxidant properties observed during fermentation with *L. plantarum*, a Partial Least Squares Regression (PLSR) analysis was employed. This supervised multivariate approach integrated the 34 quantified non-volatile compounds (5′-nucleotides, free amino acids, organic acids, and reducing sugars) as the predictive *X* matrix and the antioxidant responses (TPC, FRAP, and CUPRAC) as the dependent *Y* matrix.

**Figure 2:**
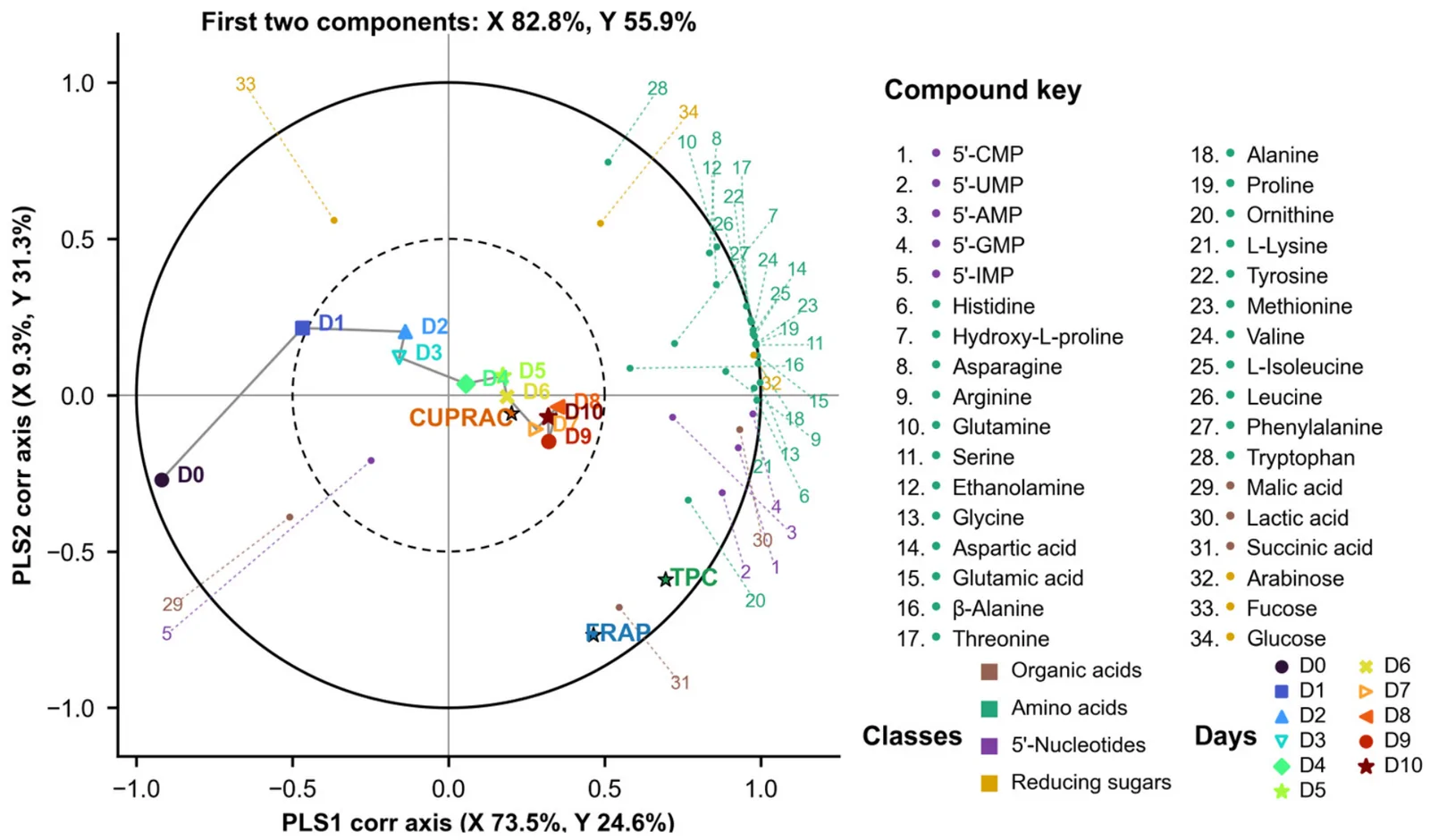
Partial Least Squares Regression (PLSR) correlation biplot illustrating the temporal changes in the LMM non-volatile metabolome and its relationship with antioxidant capacity during *L. plantarum* fermentation. TPC: total phenolic content; CUPRAC: cupric reducing antioxidant capacity; FRAP: ferric reducing antioxidant power.

Figure 2 presents the PLSR correlation biplot, which shows that fermentation time was the dominant factor driving the integrated non-volatile biochemical changes. 

The day-averaged PLSR trajectory and correlation biplot demonstrated that fermentation time was the principal factor in the non-volatile biochemical evolution of the samples. The first two latent components explained 82.8% of the variance in the non-volatile matrix (X) and 55.9% of the antioxidant response variance (Y). Supplementary PLSR projections for components 3 and 4 were also examined (Figure S2), explaining an additional 9.4% of X variance and 24.3% of Y variance and increasing the cumulative explained variance to 92.2% for X and 80.1% for Y by PLS4. These supplementary components captured secondary response-specific variation but did not alter the primary PLS1-PLS2 interpretation, in which fermentation time dominated the non-volatile biochemical trajectory. The horizontal PLS1 axis, accounting for 73.5% of the X variance, primarily captured the chronological remodelling of the matrix from a precursor-rich baseline to a mature fermented state. The negative PLS1 region isolated the unfermented Day 0 baseline, driven heavily by precursor compounds such as malic acid (29). Univariate analysis confirmed this separation, showing malic acid started at a significantly higher baseline concentration on Day 0 (1.391 ± 0.14 mg/L) and was rapidly consumed, decreasing to below the LOD (0.415 mg/L) by Day 3 (Table S1; values below the LOQ are semi-quantitative and are reported for trend indication only). Conversely, the progressive shift in later-stage samples (Days 4–10) to the positive PLS1 side was defined by the steady accumulation of fermentation-derived products. This was most prominent in the substantial production of lactic acid (30), a dynamic consistent with the fermentative metabolism of the lactic acid bacterial population [23], which increased from 1.310 ± 0.28 mg/L on Day 0 to 41.580 ± 1.36 mg/L by Day 3 and reached a maximum of 112.077 ± 3.73 mg/L on Day 9 (Table S1; values for Days 2–10 exceeding the upper calibration limit of 20 mg/L were back-calculated from a 20-fold diluted re-analysis), alongside an increase in the free amino acid pool, including L-glutamic acid (15), which accumulated significantly from 2.713 µmol/L (Day 0) to 21.429 µmol/L by Day 2 (Table S2).

While PLS1 delineated the linear timeline of metabolite accumulation, the vertical PLS2 axis, which accounted for a substantial 31.3% of the antioxidant (Y) variance, effectively separated the specific developmental changes with fermentation. Fermentation samples from Days 1 to 3 were characterised by an early “burst” phase associated with the transient release of specific metabolites. For instance, the reducing sugars fucose (33) and glucose (34) had high positive loadings along PLS2. Fucose significantly increased from its Day 0 baseline (0.903 ± 0.14 µmol/L) to an absolute peak on Day 1 (1.367 ± 0.33 µmol/L) before declining significantly by Day 2 (0.976 ± 0.22 µmol/L) (Table S4). Concurrently, there was an initial release of specific amino acids, such as L-threonine (17), which increased significantly from 2.081 µmol/L at Day 0 to 11.183 µmol/L within 48 h (Table S2). 

Following a transitional phase (Days 4–6), the late-stage samples (Days 7–10) formed a late-stage cluster along the positive axis of PLS1. The samples aligned with the total phenolic content (TPC) and ferric reducing antioxidant power (FRAP) vectors. The statistical data corroborated this dynamic, non-linear behaviour: TPC experienced a significant initial decrease from 1.773 ± 0.02 mg GAE/g on Day 0 to 1.480 ± 0.01 mg GAE/g on Day 1, before increasing significantly to 1.926 ± 0.01 mg GAE/g by Day 3 (Table S5). FRAP exhibited a similar pattern. Furthermore, succinic acid (31) was positioned closest to the TPC and FRAP direction in this quadrant, supporting its strong association with the late fermentation stage and the more antioxidant-active samples. Ultimately, these multivariate and univariate results collectively confirm that the late fermentation period generated an integrated non-volatile matrix characterised by stronger antioxidant responses and a coordinated structural remodelling. This increase in TPC and FRAP antioxidant capacity aligns with established evidence that phenolic compounds are primary contributors to the antioxidant properties of edible fungi [24]. This enhancement is nutritionally significant, as natural antioxidants can mitigate oxidative stress and associated chronic diseases by protecting cellular components from free radical damage [25]. Moreover, these fermentation-derived antioxidants offer a safer and more consumer-acceptable alternative to synthetic additives such as BHA and BHT [26].

Finally, to identify the most significant individual contributors to these predictive models, variable importance in projection (VIP) scores were calculated (Figure S1). The VIP profiles indicate that the enhanced bioactivity is not driven by a single metabolite, but by coordinated changes across chemical families. For TPC, succinic acid and tryptophan exhibited the highest VIP values, followed by 5′-CMP, glutamine, and asparagine, suggesting that phenolic responses develop in tandem with microbial acidification and nitrogenous matrix turnover. Similarly, the FRAP model was strongly driven by tryptophan, succinic acid, and a broad array of amino acids and nucleotides, linking ferric-reducing capacity closely to the amino acid and nucleotide remodelling of the late stages. The CUPRAC model displayed a wider profile that notably included fucose alongside amino acids and organic acids, corroborating its broader reductant pool observed in the biplot. The strong correlation between these low-molecular-weight metabolites and antioxidant activity is structurally sound. Free aromatic amino acids such as tryptophan act as potent electron donors and radical scavengers [27,28], while fermentation-derived organic acids such as succinic acid were also strongly associated with the late-stage antioxidant-active quadrant in the present model. Furthermore, the microbial breakdown of the matrix during fermentation releases a synergistic pool of low-molecular-weight reductants, including soluble nucleotides and free amino acids, which may collectively contribute to the enhanced reducing power of the fermented product [29].

### 3.8. Predictive Modelling of Antioxidant Capacity Using Sparse Elastic-Net Regression

To validate the associations identified in the PLSR analysis and evaluate their predictive relevance, an assay-specific elastic-net regression was applied to the integrated non-volatile dataset. This sparse modelling approach serves to isolate a reduced subset of compounds that maintain the strongest and most stable associations with total phenolic content (TPC) and ferric reducing antioxidant power (FRAP). By applying a fixed penalty (α = 0.05, l1_ratio = 0.90), the model successfully identified stable model-supported variables for the validated TPC and FRAP responses associated with the functional transformation of the LMM matrix.

#### 3.8.1. Model Performance and Internal Validation

**Figure 3:**
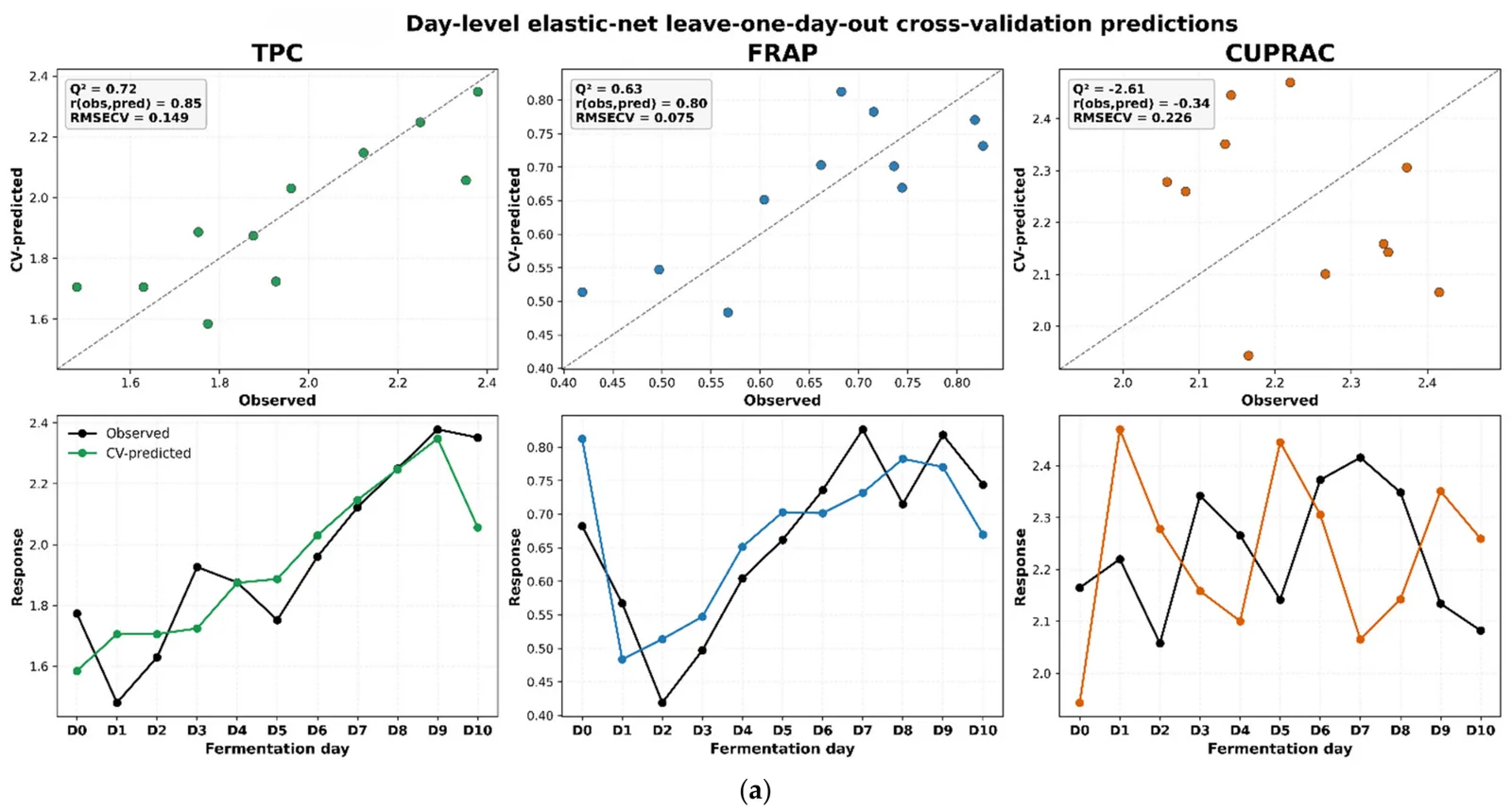

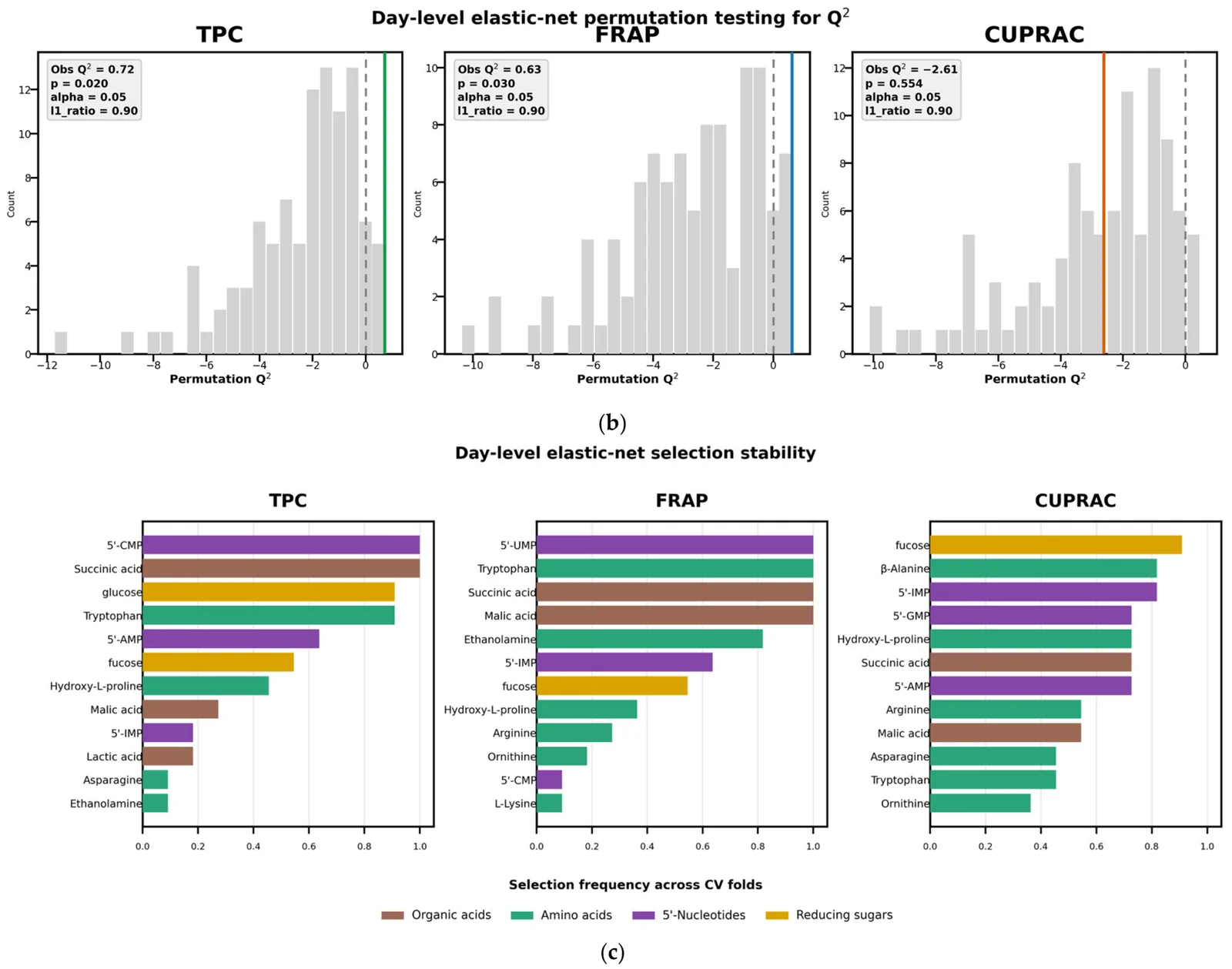
Internal validation and predictive stability of assay-specific elastic-net models showing (**a**) cross-validation performance, (**b**) permutation testing, and (**c**) frequency plots that highlight the metabolites most consistently retained across model folds, primarily involving organic acids and 5′-nucleotides. In the upper panels of (**a**), the dashed diagonal line represents the line of perfect agreement (*y* = *x*). In the lower panels of (**a**), black denotes the observed response and the coloured line denotes the cross-validated predicted response for the corresponding assay. In (**b**), the coloured vertical line marks the observed Q^2^ of the actual model. CUPRAC is included to show model validation outcome but was not used for downstream interpretation of associations because of its negative Q^2^.

Internal validation demonstrated that the performance of the elastic-net models was strongly assay-dependent (Figure 3). Using day-level leave-one-day-out cross-validation, the TPC model achieved a Q^2^ of 0.72, a Pearson correlation r(obs,pred) = 0.85 between observed and cross-validated predicted values, and an RMSECV of 0.149. This indicates good predictive relevance and close agreement between the observed and predicted values. The FRAP model was similarly supported, yielding a Q^2^ of 0.63, an r(obs,pred) = 0.80, and an RMSECV of 0.075, which demonstrates moderate to good predictive relevance. In contrast, the CUPRAC model performed poorly (Q^2^ = −2.61, r(obs,pred) = −0.34, RMSECV = 0.226), indicating that this fixed-penalty linear model predicted held-out days less accurately than the mean response.

Permutation testing further corroborated these conclusions. The observed Q^2^ values for TPC and FRAP were significantly better than those obtained under random day-level permutations (*p* = 0.020 and *p* = 0.030, respectively). Conversely, the CUPRAC model was not significant (*p* = 0.554). Therefore, the measured non-volatile compound matrix provided meaningful and non-random predictive information for TPC and FRAP, but not for CUPRAC under the present elastic-net formulation. Consistent with the PLSR validation results, the sparse linear modelling framework captured TPC most strongly, FRAP moderately, and CUPRAC poorly. Because the CUPRAC elastic-net model showed negative predictive relevance and a non-significant permutation result, subsequent elastic-net interpretation was restricted to the validated TPC and FRAP models. CUPRAC outputs are retained only for transparency and should be considered exploratory rather than biologically interpretable.

#### 3.8.2. Interpretation-Focused Coefficient Profiles

**Figure 4:**
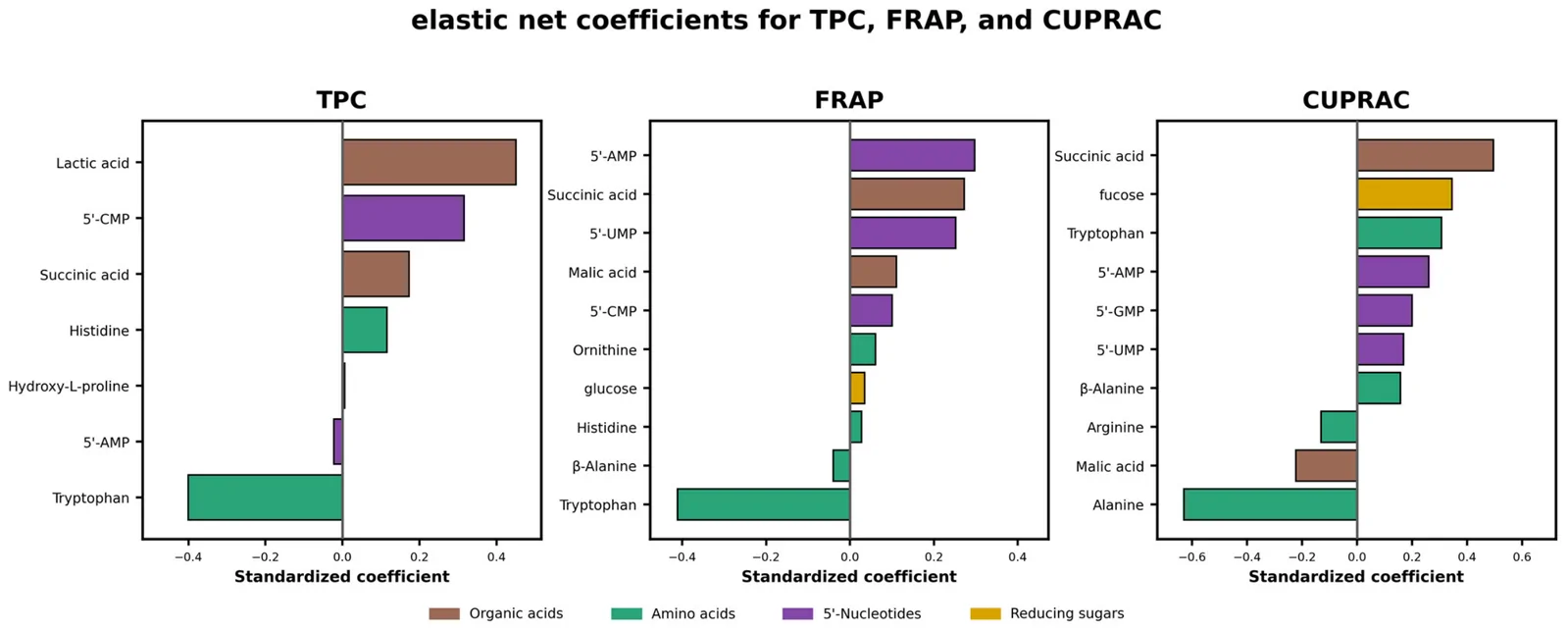
Standardised coefficient bar plots illustrate the specific non-volatile compounds retained by the regularised models and the direction of their associations with each assay. CUPRAC is shown for completeness only and should not be used for interpretation of associations because its elastic-net model failed validation.

The full-data standardised coefficient bar plots (Figure 4) provide the clearest summary of the specific compounds retained by the regularised models and the direction of their associations for the validated TPC and FRAP models. For TPC, the strongest positive coefficients were observed for lactic acid, 5′-CMP, succinic acid, and histidine, while tryptophan showed a strong negative coefficient and 5′-AMP contributed slightly negatively. This profile indicates that the TPC model was driven primarily by a combination of fermentation-related organic acids and selected nitrogen-containing compounds.

For FRAP, the dominant positive coefficients were 5′-AMP, succinic acid, 5′-UMP, malic acid, and 5′-CMP, with additional minor positive contributions from ornithine, glucose, and histidine. Tryptophan and β-alanine contributed negatively. Compared with TPC, the FRAP model exhibited a somewhat stronger nucleotide component alongside the continued importance of the organic acid block. The CUPRAC coefficient profile was considerably more mixed and should be interpreted cautiously due to the model’s poor validation. Although the CUPRAC panel is retained in Figure 4 for completeness, these coefficients are not used for interpretation of associations because the CUPRAC elastic-net model failed internal validation.

#### 3.8.3. Coefficient Paths and Selection Stability

**Figure 5:**
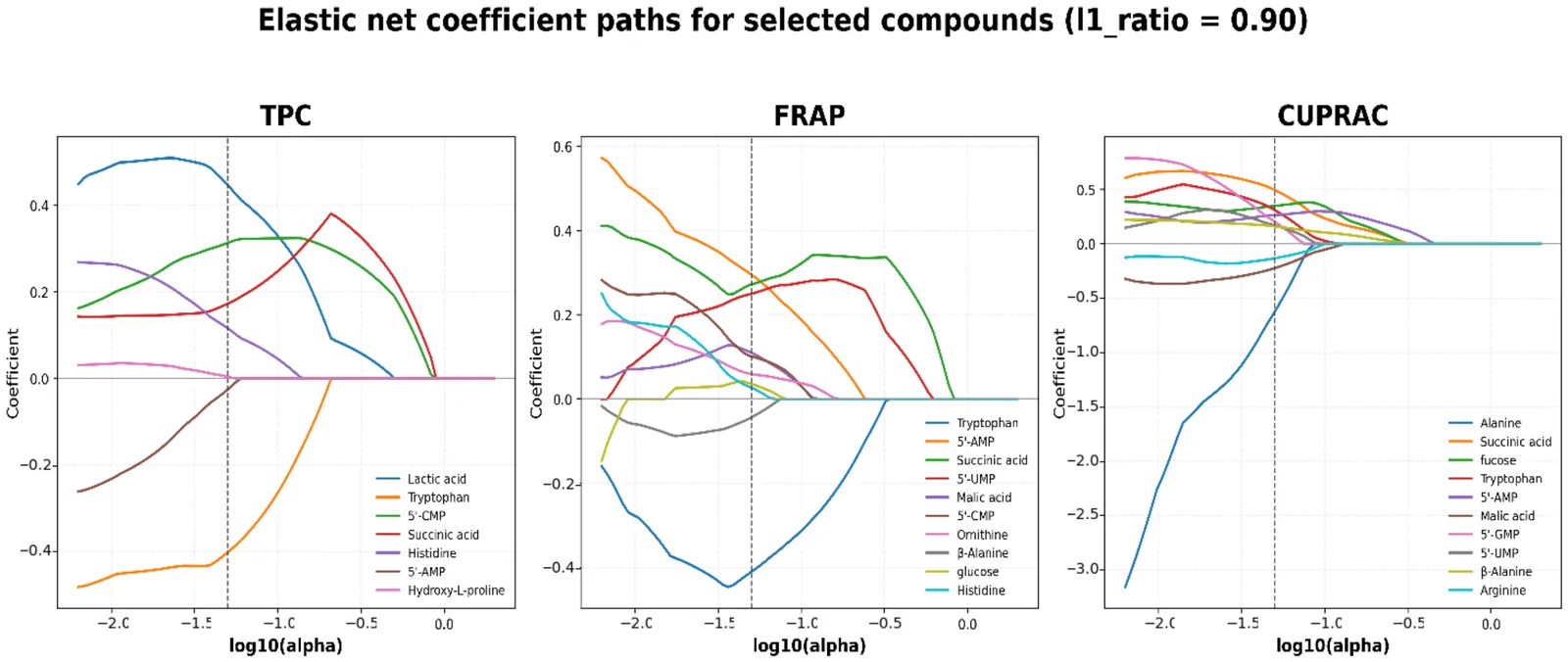
Elastic-net coefficient paths: Visualisation of coefficient shrinkage and sign persistence across a predefined α path at a fixed l1_ratio of 0.90. CUPRAC paths are shown only as exploratory output because the CUPRAC model did not pass internal validation. The vertical dashed line indicates the fixed penalty setting (α = 0.05) applied across the models. CUPRAC paths are shown only as exploratory output because the CUPRAC model did not pass internal validation.

The coefficient-path plots (Figure 5) and selection-stability analysis (Figure 3c) provide supplementary visualisations of which compounds were retained consistently as the regularisation penalty was varied and as days were omitted during cross-validation. This approach allows us to trace the shrinkage of coefficients across a predefined α path [30], demonstrating the variable selection capabilities inherent to the elastic-net methodology [31]. 

For TPC, 5′-CMP, succinic acid, glucose, and tryptophan were selected with the highest stability across folds, while 5′-AMP, fucose, and hydroxy-L-proline exhibited intermediate retention. For FRAP, 5′-UMP, tryptophan, succinic acid, and malic acid were among the most stable variables. However, stability alone should not be over-interpreted as evidence of predictive usefulness. Notably, the CUPRAC model selected several variables, such as fucose, β-alanine, and 5′-IMP, relatively consistently despite its poor Q^2^ and non-significant permutation test. This indicates that CUPRAC variable retention reflects repeated selection by the regularisation procedure, but was not a reliable predictive support. Accordingly, CUPRAC stability and coefficient patterns are shown only for transparency, and interpretation of associations was restricted to the validated TPC and FRAP models.

#### 3.8.4. Day-Wise Contribution Heatmap

**Figure 6:**
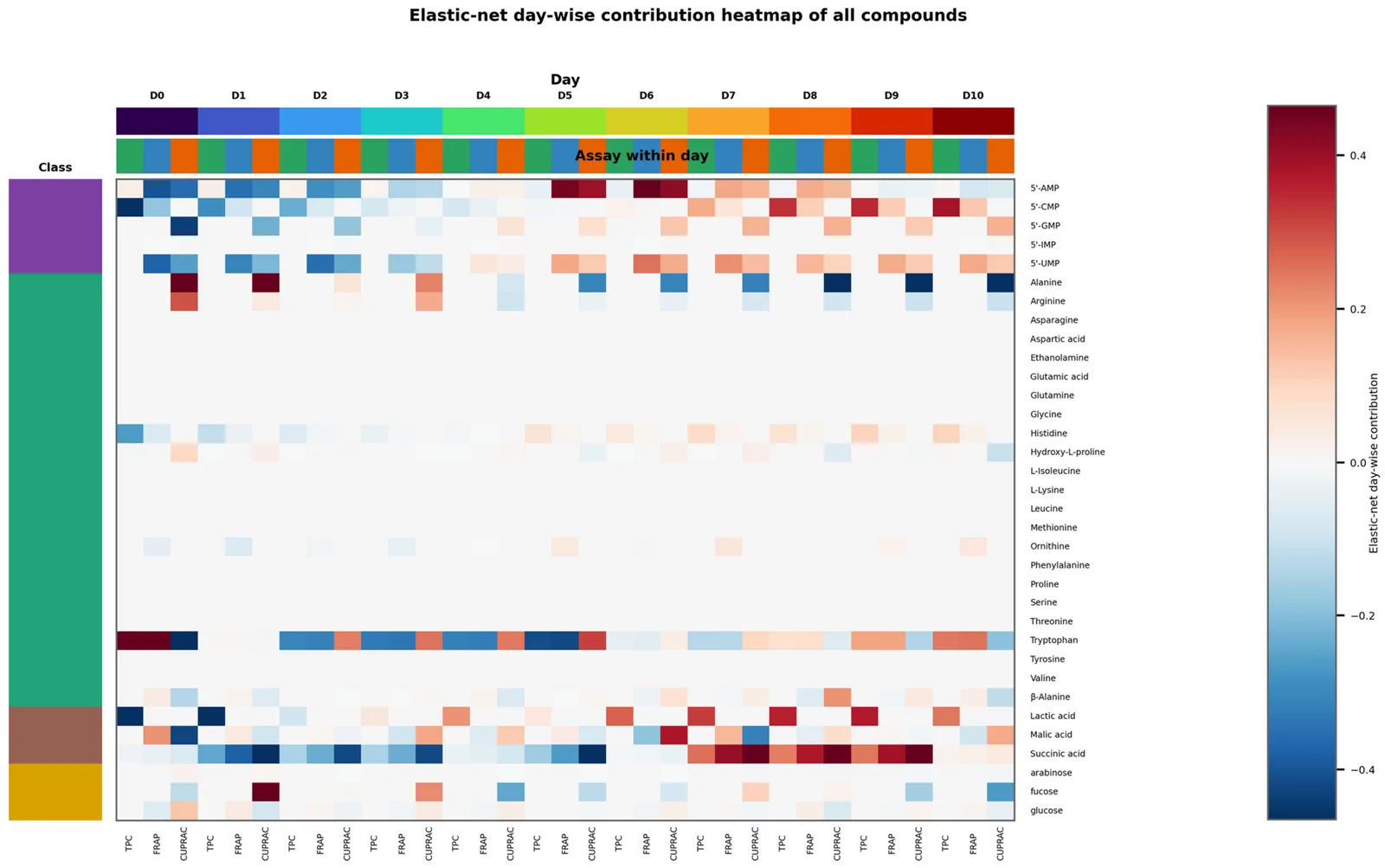
Model-derived day-wise contribution heatmap: A descriptive representation of the influence of individual compounds across the 10-day fermentation period. Each cell represents the product of the full-model coefficient and the standardised compound value. CUPRAC-related patterns should be interpreted cautiously because the CUPRAC elastic-net model failed validation.

Finally, the model-derived day-wise contribution heatmap (Figure 6) serves as a descriptive representation of the evolving influence of individual compounds across the 10-day fermentation period. Unlike a standard Pearson correlation heatmap, each cell here represents the product of the full-model coefficient and the corresponding standardised compound value at a given day. As expected for a regularised model where many coefficients are shrunk to zero, most compound contributions remained close to zero. The visible day-wise structures were concentrated in a limited subset of features, including selected 5′-nucleotides, succinic acid, lactic and malic acids, glucose, fucose, and tryptophan. Because the sign and magnitude of the contribution depend on both the coefficient direction and the standardised compound value relative to the global mean, the same compound can contribute positively on certain days and negatively on others. Consequently, this heatmap effectively illustrates the dynamic expression of the sparse elastic-net model across fermentation time. Therefore, this heatmap should be interpreted descriptively, with interpretation of associations focused on the validated TPC and FRAP models. CUPRAC-related contribution patterns are retained only as exploratory visual information.

### 3.9. Evaluation of Equivalent Umami Concentration (EUC) and Taste Activity Values (TAVs)

To evaluate the sensory significance of the biochemical shifts observed during fermentation, the synergistic umami potential and individual taste contributions were modelled. The umami intensity of mushrooms is significantly enhanced by the synergy between umami-contributing free amino acids (Glu and Asp) and 5′-ribonucleotides (5′-GMP and 5′-IMP). This interaction occurs at the T1R1/T1R3 taste receptors, where the presence of nucleotides exponentially increases the binding affinity of glutamic acid [32].

Based on the quantified concentrations and the synergy formula established by Yamaguchi et al. [20] (pp. 846–849), the Equivalent Umami Concentration (EUC) of the LMM increased by an estimated 24-fold over the 10-day period, increasing from 0.27 g MSG/100 g at Day 0 to 6.49 g MSG/100 g at Day 10, as seen in Table 1. While the concentration of 5′-GMP remained relatively stable, the significant surge in free glutamic acid (*p* < 0.0001) provided the necessary substrate for this synergistic increase. The literature strongly supports this mechanism; while nucleotides like 5′-GMP do not directly activate umami receptors on their own, they are known to amplify the umami sensation triggered by glutamate by up to eightfold [33]. The efficacy of our fermentation protocol is further validated by recent findings that *L. plantarum* is uniquely effective among microorganisms at increasing free amino acids and 5′-nucleotides to produce intense umami flavours in mushroom matrices [34]. Beyond umami generation, fermentation may also contribute to mitigating the inherent triterpenoid-driven bitterness of *G. lucidum*. Based on the modelled TAVs, the sub-threshold accumulation of 5′-AMP and the sweet amino acid alanine could provide competitive antagonism at bitter taste receptors and cross-modal masking, respectively. However, neither triterpenoid content nor actual bitter taste perception was measured in this study, and sensory evaluation is required to confirm this hypothesis. 

To determine which metabolites contributed directly to the perceived flavour profile, Taste Activity Values (TAVs) were calculated (Table 2) using thresholds defined by Mau [21]. A TAV > 1.0 indicates that a compound is present at a concentration that is sensorially perceptible. By Day 10, both 5′-GMP (TAV = 2.82) and glutamic acid (TAV = 1.27) exceeded this threshold. The strong TAV for 5′-GMP highlights its role as a potent flavour enhancer, working in tandem with the primary umami substrate, glutamic acid.

These results suggest a possible dual-action mechanism for mitigating the inherent bitterness of *G. lucidum*. While 5′-AMP (TAV = 0.15) and alanine (TAV = 0.79) did not cross the absolute perception threshold, their accumulation may still contribute to the overall flavour profile. Sub-threshold 5′-AMP has been reported to act as a competitive antagonist at human bitter taste receptors (T2Rs) [35] and the significant rise in sweet-tasting metabolites, notably alanine (*p* < 0.0001), may contribute to the masking of bitter notes, consistent with the recognised role of sweet-tasting compounds as bitter-masking agents [36]. Whether these mechanisms translate into a perceived reduction in bitterness in fermented LMM requires sensory confirmation. Collectively, these model-based findings suggest that *L. plantarum* fermentation may remodel the LMM from a raw, medicinal matrix into a more balanced, savoury food base, although confirmation of consumer acceptability would require formal sensory evaluation.

### 3.10. Principal Component Analysis of the Volatile Compounds of LMM During Fermentation

**Figure 7:**
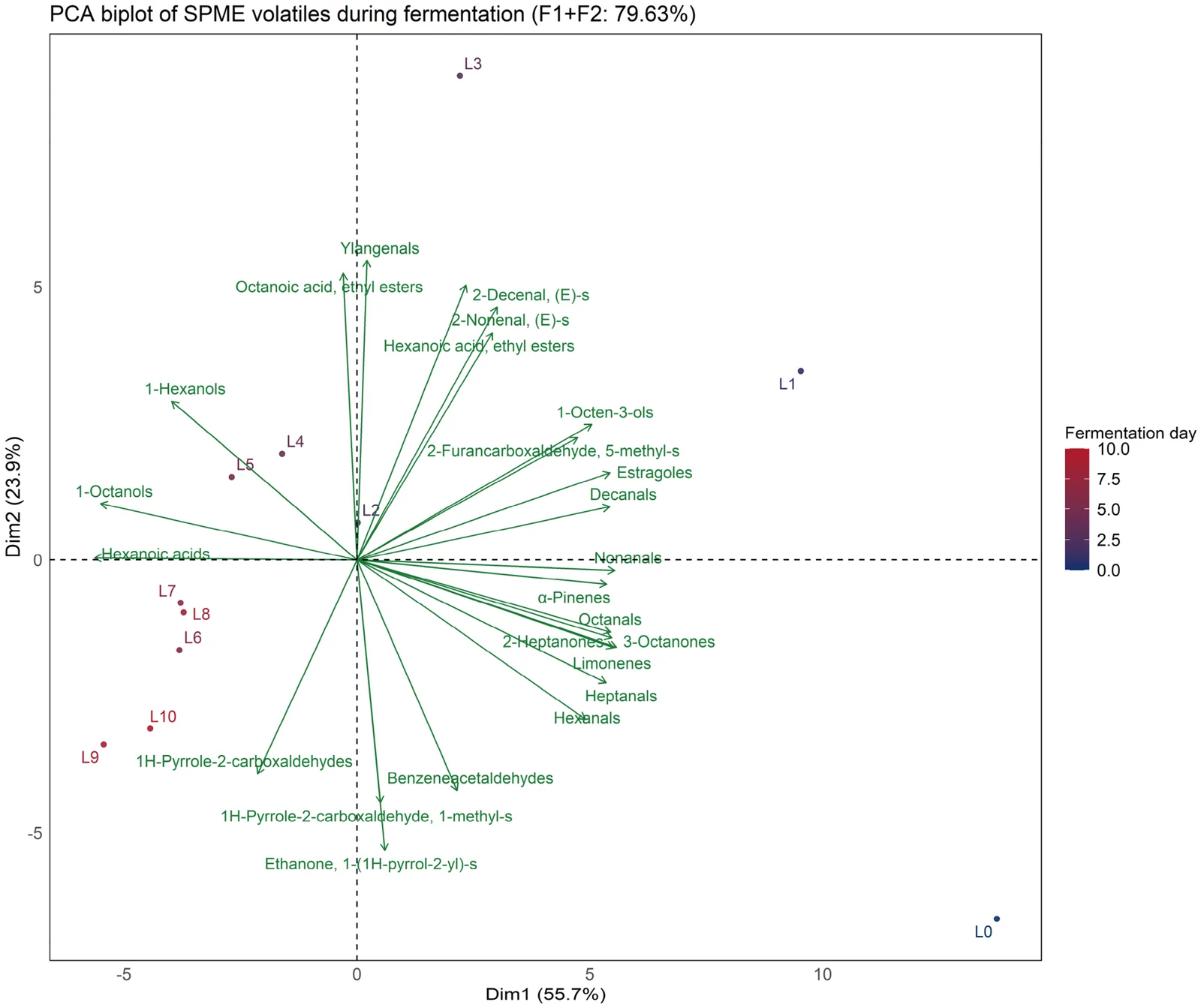
Principal Component Analysis (PCA) biplot of the volatile compounds and LMM samples throughout the fermentation process. The volatile compounds discussed in the text are labelled; samples are coloured by fermentation day.

Figure 7 presents the Principal Component Analysis (PCA) biplot of volatile compounds in LMM powder samples across the L. plantarum fermentation process (Days 0–10). The first two principal components (F1 and F2) collectively explained 79.63% of the total variance (F1: 55.70%; F2: 23.93%), effectively capturing the dominant trajectory of volatile evolution during fermentation. All volatile compounds were tentatively identified (Section 2.8). Relative abundances are expressed as the percentage of the total peak area of all identified compounds, as no internal standard was used. The PCA was performed on the mean-centred and unit-variance-scaled peak areas of the volatile compounds using the FactoMineR package in RStudio (Posit, PBC, USA).

F1 accounted for 55.70% of the total variance and primarily reflected the progression of fermentation time. The sample L0 was positioned at the extreme positive end of F1, characterised by high relative abundances of long-chain aldehydes and ketones such as nonanal, decanal, octanal, 2-heptanone and 3-octanone, compounds typically derived from lipid oxidation or thermal degradation during drying. The early presence of these aldehydes and ketones is a well-documented result of initial lipid degradation, specifically the hydrolysis and oxidation of polyunsaturated fatty acids [37]. From Day 1 onwards, their relative abundances declined progressively (e.g., nonanal from 1.3% at Day 0 to 0.7% at Day 10), consistent with the leftward migration of samples along F1 from Day 0 to Day 10.

Samples from mid-fermentation (Days 3–5) were positioned towards the negative end of F1 and the positive end of F2, reflecting the transient peak of several microbially derived volatiles. Notably, 1-octen-3-ol, which was a key C-8 “mushroom alcohol” with a characteristic savoury aroma [38], reached its highest relative abundance at Day 3 (13.6%) before declining towards Day 10 (5.4%). Similarly, ethyl hexanoate and ethyl octanoate peaked at Day 3 (0.62% and 0.60%, respectively) and decreased thereafter, indicating that ester formation was most active during mid-fermentation rather than in the late stage. The distinct positioning of the L3 sample on the positive F2 axis was associated with elevated relative abundances of phenolic derivatives, furanic compounds and unsaturated aldehydes such as (E)-2-nonenal and (E)-2-decenal, many of which arise from Maillard reactions, Strecker degradation or selective microbial β-oxidation.

In contrast, late fermentation (Days 6–10) was characterised by the accumulation of hexanoic acid (from 3.0% at Day 0 to 12.4% at Day 10) and saturated alcohols such as 1-hexanol and 1-octanol, while the relative abundances of esters and 1-octen-3-ol declined from their mid-fermentation peaks. The concurrent increase in hexanoic acid and decline of esters in the late stage suggests that ester formation was favoured during the active growth phase and diminished as fermentation progressed, rather than a continuous esterification of free fatty acids. Samples with negative F2 scores (from L6 to L10) were characterised by elevated relative abundances of benzeneacetaldehyde and pyrrole derivatives (e.g., 1H-pyrrole-2-carboxaldehyde and 1-(1H-pyrrol-2-yl)ethanone), consistent with the progressive formation of Maillard and Strecker degradation products as fermentation proceeded.

The overall trajectory of samples formed a clear arc from L0 to L10 in the PCA space, indicating a continuous and directional transformation of the volatile profile: early fermentation (Days 0–2) was dominated by lipid-derived aldehydes and ketones, mid-fermentation (Days 3–5) by mushroom alcohols, esters, furans, phenolics and unsaturated carbonyls, and late fermentation (Days 6–10) by free fatty acids and saturated alcohols. These phased changes in relative abundance highlight the dynamic interplay between residual substrate availability and microbial metabolic activity (such as esterases and alcohol dehydrogenases) over time. Notably, aldehydes such as hexanal and heptanal, characterised by green, grassy aromas, were prominent in the early samples but declined markedly over time, likely reflecting their microbial reduction to the corresponding alcohols. While this section describes the global trends in volatile dynamics, it does not quantify the contribution of individual compounds to specific functional attributes.

### 3.11. LMM as a Natural Multi-Functional Flavour Enhancer

Beyond its nutritional enrichment, the fermented LMM developed in this study shows potential as a natural alternative to synthetic food additives. The food industry faces significant pressure to replace synthetic antioxidants, such as butylated hydroxyanisole (BHA) and butylated hydroxytoluene (BHT), as well as exogenous flavour enhancers such as monosodium glutamate (MSG). The results demonstrate that *L. plantarum* fermentation generates a matrix rich in natural antioxidants and synergistic umami compounds, which could support clean-label formulations when used as a food ingredient. Furthermore, the combination of model-supported umami enhancement and the proposed bitterness-mitigation mechanism suggests that fermented LMM could support sodium reduction by contributing umami intensity and masking off-notes in low-sodium formulations. However, because neither sensory evaluation nor triterpenoid quantification was performed in this study, these applications remain proposed rather than demonstrated and warrant sensory and consumer studies. If confirmed, this multi-functionality would position fermented LMM as a high-value, sustainable ingredient for the plant-based and functional food sectors.

## 4. Limitations

The findings of this study should be interpreted in light of several methodological limitations. First, because no time-matched uninoculated control was included, the unfermented Day 0 (L0) sample served as the baseline. Although the indigenous microbiota of LMM was present and active during fermentation (Figure 1), the relative contributions of the inoculated strain and the indigenous microorganisms to the observed changes could not be strictly separated. Second, the bitterness-related conclusions mentioned are model-based inferences derived from taste-active metabolite concentrations (TAVs) and published taste mechanisms. Neither triterpenoid content nor actual bitter taste perception was measured, and sensory evaluation is required to confirm these hypotheses. Third, the elastic-net models were based on a limited number of effective time points (*n* = 11 days) relative to the number of predictors (*n* = 34) and should therefore be regarded as exploratory rather than confirmatory. Fourth, metabolite concentrations are reported on a wet (fresh) weight basis, as the fermented samples were extracted without prior drying. Finally, volatile compounds were tentatively identified by mass spectral library matching and retention indices without confirmation by authentic standards. 

Several analytical limitations should be noted. First, after re-fitting of the organic acid calibration curves (see Section 2.6), the LOD/LOQ values are 0.415/1.258, 0.094/0.285 and 0.292/0.884 mg/L for malic, lactic and succinic acids, respectively. Malic acid was quantifiable on one day and below the LOQ (or LOD) on the remainder; succinic acid was quantifiable on seven days; and values below the LOQ are reported as semi-quantitative. Lactic acid concentrations exceeded the upper calibration limit (20 mg/L) on Days 2–10, and these values were back-calculated from a 20-fold diluted re-analysis (maximum 112.1 mg/L, i.e., 5.6-fold), as disclosed in the footnote of Table S1. Second, after re-fitting of the 5′-nucleotide calibration curves by weighted (1/x) least squares, 5′-GMP and 5′-UMP were quantifiable on all eleven sampling days, whereas 5′-CMP was quantifiable on eight days (from Day 3 onward), 5′-AMP was quantifiable on two days, and 5′-IMP was below the LOD throughout and is reported as not detected. Because nucleotide concentrations are low, the EUC and TAVs derived from them should be read as model-based estimates rather than fully validated quantitative results. The calibration window for the 5′-nucleotides (7.8–1000 µmol/L) was designed prior to knowledge of the sample concentrations and lies partly above the range subsequently observed (0.14–23.0 µmol/L, day means); 107 of 165 individual readings fell below the lowest calibrator. Although the weighted regression compensates in part for this mismatch, the nucleotide data should still be regarded as semi-quantitative. In addition, metabolites were extracted into absolute methanol (Section 2.2.2); the extraction recovery for the highly polar 5′-mononucleotides was not separately determined and may have been incomplete, which could contribute to the low nucleotide concentrations observed. Third, MRS plate counts reflect the total lactic acid bacterial population of the fermentation matrix and do not confirm that the enumerated colonies correspond to the inoculated *L. plantarum* strain.

## 5. Conclusions

Controlled fermentation with *L. plantarum* is an effective bioprocessing strategy for the functional transformation of Lingzhi mushroom mycelium (LMM). Over a 10-day period, the LMM matrix underwent significant biochemical remodelling, highlighted by a marked increase in free glutamic acid and key 5′-nucleotides, resulting in an estimated 24-fold increase in EUC. Concurrently, the presence of 5′-AMP below its taste threshold provides a plausible mechanistic basis for potential antagonistic bitterness masking, which could contribute to an improved sensory profile of the fermented ingredient; sensory confirmation is warranted. Furthermore, PLSR and elastic-net modelling supported exploratory associations between late-stage fermentation (Days 7–10), enhanced TPC/FRAP antioxidant responses, microbial generation of succinic acid, and nitrogenous matrix turnover, while CUPRAC was more heterogeneous and less well captured by the sparse elastic-net model. Overall, this study establishes that targeted *L. plantarum* fermentation can upgrade raw LMM into a bioactive ingredient with substantially enhanced umami and antioxidant potential, which may support its use as a natural flavour enhancer.

## Figures and Tables

**Table 1:** Equivalent Umami Concentration (EUC) and contributing umami metabolites in Lingzhi mushroom mycelium (LMM) during a 10-day fermentation with *L. plantarum*.

Day	Glu (µmol/L)	Asp (µmol/L)	5′-GMP (µmol/L)	5′-IMP (µmol/L)	Calculated EUC (g MSG/100 g)
0	2.713 ± 0.553	0.827 ± 0.274	6.364 ± 0.766	n.d.	0.27 ± 0.08
1	10.359 ± 1.226	5.358 ± 0.662	7.419 ± 0.666	n.d.	1.21 ± 0.19
2	21.429 ± 2.181	9.064 ± 0.437	7.619 ± 0.594	n.d.	2.55 ± 0.26
3	19.553 ± 1.341	7.560 ± 1.074	8.435 ± 0.453	n.d.	2.57 ± 0.23
4	28.003 ± 0.839	10.567 ± 0.304	9.037 ± 0.752	n.d.	3.93 ± 0.25
5	31.522 ± 0.503	12.679 ± 0.523	9.119 ± 0.144	n.d.	4.47 ± 0.10
6	35.933 ± 0.996	13.045 ± 0.645	9.459 ± 0.176	n.d.	5.27 ± 0.11
7	36.835 ± 1.087	14.060 ± 0.223	9.660 ± 0.128	n.d.	5.53 ± 0.21
8	41.736 ± 2.564	14.678 ± 1.290	9.704 ± 0.175	n.d.	6.27 ± 0.29
9	40.754 ± 3.264	15.904 ± 1.366	9.419 ± 0.382	n.d.	5.97 ± 0.65
10	43.046 ± 3.404	16.522 ± 0.839	9.697 ± 0.397	n.d.	6.49 ± 0.52

Note: Values represent the mean concentration of umami-active amino acids and 5′-nucleotides used to calculate the synergistic umami intensity. EUC is expressed as grams of monosodium glutamate (MSG) equivalent per 100 g of fermented LMM (wet basis). Values are presented as mean ± SD (*n* = 3). 5′-IMP was below the LOD on all sampling days and is reported as n.d.

**Table 2:** Individual Taste Activity Values (TAVs) of key flavour-active metabolites in fermented LMM at Day 10. Concentrations are presented as mean ± SD (*n* = 3); TAVs were calculated from the mean concentrations of three biological replicates and are presented as single values. Where nucleotide concentrations fell below the LOQ, the derived EUC and TAVs are presented as indicative.

Compound	Class	Concentration (mg/100 g)	Standard Threshold (mg/100 g)	TAV
5′-GMP	5′-Nucleotide (Umami)	35.2 ± 1.4	12.5	2.82
Glutamic acid	Free Amino Acid (Umami)	63.3 ± 5.0	50	1.27
Alanine	Free Amino Acid (Sweet)	47.4 ± 4.0	60	0.79
5′-AMP	5′-Nucleotide (Masking)	7.45 ± 1.94	50	0.15

Note: TAV = concentration/threshold. A TAV > 1.0 indicates that the compound contributes significantly and directly to the perceived taste profile. Threshold values were sourced from Mau [21]. Concentrations are expressed per 100 g of fermented LMM (wet basis).

## Data Availability

The original contributions presented in this study are included in the article/Supplementary Material. Further inquiries can be directed to the corresponding author.

## Supplementary Materials

The following supporting information can be downloaded at https://www.mdpi.com/article/10.3390/antiox15091196/s1. Table S1: Changes in organic acids of LMM powder under different fermentation times (mg/L); Table S2: Changes in the free amino acid content during fermentation process from Day 0 to Day 10 (µmol/L); Table S3: Changes in the 5′-nucleotide content during the fermentation process from Day 0 to Day 10 (µmol/L); Table S4: Changes in reducing sugar content during fermentation (µmol/L); Table S5: Change in TPC and antioxidant activities during fermentation (TPC: mg gallic acid equivalent/g; CUPRAC and FRAP: mg ascorbic acid equivalent/g); Table S6: Volatile compounds tentatively identified in fermented LMM and their relative abundances during fermentation; Figure S1: Variable importance in projection (VIP) scores for individual PLSR models of total phenolic content (TPC), ferric reducing antioxidant power (FRAP), and cupric reducing antioxidant capacity (CUPRAC); Figure S2: PLS3–PLS4 correlation biplot of the PLSR model applied to the day-averaged non-volatile matrix; Figure S3: PCA biplot of all identified volatile compounds and LMM samples during fermentation. References [39,40,41,42,43,44,45,46,47,48] are cited in Supplementary Materials.
