# Supplementary material for: Biochemical and Flavour Profile of Lingzhi (Ganoderma lucidum) Mycelium Fermented by Lactiplantibacillus plantarum: A Dual-Modelling Approach to Umami Synergy and Antioxidant Capacity
Source: Antioxidants (Basel). 2026 Sep 18;15(9):1196. doi: 10.3390/antiox15091196 (PMC13603365; doi:10.3390/antiox15091196)
Supplement: Supplementary file 1 [file antioxidants-15-01196-s001.zip › antioxidants-4463582-supplementary.pdf]

L. plantarum can metabolise substrates in mushrooms, thereby increasing the content of organic acids [1, 2]. Organic acids, especially citric, succinic and malic acids, may have protective effects against various diseases due to their antioxidant activity [3]. At the same time, the combined action of organic acids greatly contribute to the formation of the unique flavour of mushrooms [4]. In this experiment, seven organic acids were determined including tartaric acid, pyruvic acid, malic acid, lactic acid, citric acid, fumaric acid and succinic acid. However, only three organic acids including malic, lactic, and succinic acid were detected in the fermented LMM.

Table S1. Changes in organic acids of LMM powder under different fermentation time (mg/L).

Time (Day)	Malic acid (mg/L)	Lactic acid (mg/L)	Succinic acid (mg/L)
0	1.391 ± 0.14a	1.310 ± 0.28h	1.076 ± 0.07b
1	0.783 ± 0.05c	1.183 ± 0.28h	0.528 ± 0.07d
2	0.680 ± 0.09c	26.421 ± 1.69g	0.744 ± 0.08c
3	0.407 ± 0.02e	41.580 ± 1.36f	0.746 ± 0.06c
4	0.485 ± 0.14e	68.037 ± 3.10e	1.064 ± 0.06b
5	0.762 ± 0.12c	41.329 ± 4.88f	0.703 ± 0.21cd
6	0.182 ± 0.01f	83.538 ± 1.22c	1.115 ± 0.02b
7	1.172 ± 0.08b	96.570 ± 8.71b	2.089 ± 0.26a
8	0.533 ± 0.05de	108.788 ± 4.16a	2.003 ± 0.16a
9	0.673 ± 0.03cd	112.077 ± 3.73a	2.050 ± 0.14a
10	0.412 ± 0.05e	76.450 ± 3.09d	1.193 ± 0.03b
Pr > F(Model)	<0.0001	<0.0001	<0.0001
Significant	Yes	Yes	Yes
Pr > F(Day)	<0.0001	<0.0001	<0.0001
Significant	Yes	Yes	Yes

*Lactic acid concentrations on Days 2–10 exceeded the upper calibration limit of the original (undiluted) run (20 mg/L) and were obtained by extrapolation; these values were verified by re-analysis of a 20-fold diluted extract back-calculated with a dilution factor of 20, which confirmed the order of magnitude and the day-to-day trend. Values below the LOQ (malic acid 1.258 mg/L; lactic acid 0.285 mg/L; succinic acid 0.884 mg/L) are semi-quantitative and are reported for trend indication only.

As seen in Table S1, the fermentation with *L. plantarum* effectively enriches the mycelial matrix with lactic and succinic acids, a trend which correlates strongly with the microbial growth curve shown in Figure 1, while concurrently reducing the malic acid content. Lactic acid exhibited the most obvious change during fermentation, followed by succinic acid. In contrast, malic acid was the most stable, with little fluctuation. The concentration of malic acid displayed an overall declining trend from

Day 0 to 10, although fluctuations occurred during the interim, and reached its lowest point on Day 6. This pattern was consistent with the degradative action of *L. plantarum* on malic acid, which was most pronounced around Day 6, corresponding to the end of the exponential microbial growth phase [5]. In contrast, the concentration of lactic acid increased significantly over time, peaking on Day 9. This accumulation confirmed the active production of lactic acid as a central metabolic output of the fermenting organism, with the subsequent decrease on Day 10 suggesting the culmination of the active fermentation process. Similarly, the concentration of succinic acid showed a general increase, with significantly higher levels on Days 7, 8, and 9 compared to other time points. The decline on Day 10 further indicated that succinic acid biosynthesis was closely linked to the active fermentation period.

A total of 22 free amino acids and ethanolamine (23 compounds) were detected in LMM in this experiment, as shown in Table S2.

Table S2. Changes in the free amino acid content during fermentation process from Day 0 to Day 10 (μmol/L)

Time (Day)	L-histidine	Hydroxy-L-proline	Asparagine	L-arginine	Glutamine	L-serine	Ethanolamine	Glycine	L-aspartic acid	L-glutamic acid	β-alanine	L-threonine
0	0.378 ^{f}	0.044 ^{b}	1.513 ^{d}	1.491 ^{f}	1.783 ^{e}	2.851 ^{f}	0.415 ^{c}	4.378 ^{g}	0.827 ^{j}	2.713 ^{g}	0.208 ^{b}	2.081 ^{f}
1	1.262 ^{ef}	0.074 ^{ab}	5.405 ^{bc}	5.013 ^{e}	6.920 ^{d}	8.102 ^{e}	0.556 ^{bc}	8.239 ^{f}	5.358 ^{i}	10.359 ^{f}	0.238 ^{ab}	7.261 ^{e}
2	1.705 ^{de}	0.085 ^{ab}	7.305 ^{a}	6.191 ^{de}	9.951 ^{bc}	14.138 ^{d}	0.699 ^{ab}	12.733 ^{e}	9.064 ^{g}	21.429 ^{e}	0.257 ^{ab}	11.183 ^{cd}
3	1.972 ^{cde}	0.089 ^{ab}	4.114 ^{c}	2.707 ^{f}	8.493 ^{c}	12.984 ^{d}	0.630 ^{ab}	11.918 ^{e}	7.560 ^{h}	19.553 ^{e}	0.261 ^{ab}	9.781 ^{d}
4	2.338 ^{bcde}	0.084 ^{ab}	5.935 ^{abc}	9.490 ^{ab}	10.491 ^{b}	16.306 ^{c}	0.638 ^{ab}	16.020 ^{d}	10.567 ^{f}	28.003 ^{d}	0.234 ^{ab}	11.284 ^{cd}
5	3.032 ^{abc}	0.101 ^{ab}	6.001 ^{ab}	7.099 ^{cde}	13.062 ^{a}	19.224 ^{b}	0.677 ^{ab}	17.334 ^{cd}	12.679 ^{e}	31.522 ^{c}	0.261 ^{ab}	15.003 ^{a}
6	2.840 ^{abcd}	0.076 ^{ab}	6.463 ^{ab}	7.328 ^{bcd}	9.936 ^{bc}	19.282 ^{b}	0.652 ^{ab}	18.406 ^{bc}	13.045 ^{de}	35.933 ^{b}	0.286 ^{ab}	12.543 ^{bc}
7	3.443 ^{ab}	0.075 ^{ab}	6.634 ^{ab}	9.027 ^{abc}	10.605 ^{b}	20.546 ^{ab}	0.683 ^{ab}	19.230 ^{bc}	14.060 ^{cd}	36.835 ^{b}	0.273 ^{ab}	14.167 ^{ab}
8	3.164 ^{ab}	0.114 ^{a}	7.199 ^{ab}	9.076 ^{abc}	10.031 ^{bc}	22.017 ^{a}	0.776 ^{a}	21.427 ^{a}	14.678 ^{bc}	41.736 ^{a}	0.339 ^{a}	14.495 ^{a}
9	3.836 ^{a}	0.091 ^{ab}	6.056 ^{ab}	9.700 ^{a}	9.549 ^{bc}	21.501 ^{a}	0.646 ^{ab}	20.282 ^{ab}	15.904 ^{ab}	40.754 ^{a}	0.277 ^{ab}	14.170 ^{ab}
10	3.783 ^{a}	0.139 ^{a}	6.757 ^{ab}	9.681 ^{a}	9.453 ^{bc}	22.607 ^{a}	0.636 ^{ab}	21.368 ^{a}	16.522 ^{a}	43.046 ^{a}	0.214 ^{b}	15.318 ^{a}
Pr > F(Day)	<0.0001	0.352	0.000	<0.0001	<0.0001	<0.0001	0.013	<0.0001	<0.0001	<0.0001	0.456	<0.0001
Significant	Yes	No	Yes	Yes	Yes	Yes	Yes	Yes	Yes	Yes	No	Yes

	L-alanine	L-proline	L-ornithine	L-lysine	L-tyrosine	L-methionine	L-valine	L-isoleucine	L-leucine	L-phenylalanine	L-tryptophan
0	10.513 ^{f}	1.534 ^{g}	0.205 ^{ab}	0.885 ^{d}	3.321 ^{f}	0.321 ^{g}	2.818 ^{g}	2.468 ^{g}	4.253 ^{g}	3.539 ^{i}	2.645 ^{f}
1	21.001 ^{e}	5.689 ^{f}	0.178 ^{b}	1.799 ^{c}	11.433 ^{e}	2.329 ^{f}	12.372 ^{f}	9.612 ^{f}	19.841 ^{f}	11.426 ^{h}	4.215 ^{cd}
2	33.644 ^{c}	11.339 ^{d}	0.233 ^{ab}	2.251 ^{bc}	17.191 ^{d}	4.471 ^{e}	25.868 ^{e}	18.642 ^{e}	38.939 ^{e}	18.713 ^{fg}	4.813 ^{ab}
3	29.520 ^{d}	9.494 ^{e}	0.214 ^{ab}	2.323 ^{bc}	16.316 ^{d}	4.219 ^{e}	22.460 ^{e}	16.215 ^{e}	34.856 ^{e}	17.578 ^{g}	4.855 ^{ab}
4	37.208 ^{c}	12.925 ^{d}	0.251 ^{ab}	2.697 ^{b}	21.121 ^{c}	5.820 ^{d}	30.791 ^{d}	21.958 ^{d}	45.910 ^{d}	21.974 ^{ef}	4.835 ^{ab}
5	43.891 ^{b}	15.508 ^{c}	0.310 ^{a}	3.770 ^{a}	22.819 ^{bc}	6.509 ^{cd}	35.644 ^{c}	25.411 ^{c}	52.125 ^{cd}	24.474 ^{cde}	5.026 ^{a}
6	44.010 ^{b}	14.739 ^{c}	0.243 ^{ab}	3.378 ^{a}	21.985 ^{c}	6.431 ^{cd}	35.974 ^{c}	25.972 ^{c}	53.896 ^{c}	23.917 ^{de}	4.309 ^{cd}
7	44.158 ^{b}	16.245 ^{bc}	0.320 ^{a}	3.711 ^{a}	24.493 ^{abc}	7.095 ^{bc}	37.497 ^{bc}	26.975 ^{bc}	55.764 ^{bc}	26.113 ^{bcd}	4.463 ^{bc}
8	50.621 ^{a}	17.605 ^{ab}	0.254 ^{ab}	3.297 ^{a}	25.664 ^{ab}	8.155 ^{a}	41.644 ^{a}	30.805 ^{a}	64.223 ^{a}	29.539 ^{ab}	4.088 ^{cde}

9	50.648 [a]	18.058 [ab]	0.275 [ab]	3.846 [a]	24.488 [abc]	7.309 [abc]	41.580 [ab]	29.981 [ab]	61.991 [ab]	27.471 [abc]	3.900 [de]
10	53.203 [a]	19.155 [a]	0.316 [a]	3.701 [a]	27.068 [a]	7.876 [ab]	44.217 [a]	30.853 [a]	65.653 [a]	30.296 [a]	3.793 [e]
Pr > F(Day)	<0.0001	<0.0001	0.261	<0.0001	<0.0001	<0.0001	<0.0001	<0.0001	<0.0001	<0.0001	<0.0001
Significant	Yes	Yes	No	Yes	Yes	Yes	Yes	Yes	Yes	Yes	Yes

The fermentation process significantly increases the free amino acid (FAA) pool of LMM. Results confirmed that the concentration of most FAAs significantly increased ($p < 0.05$) over the 10-day period. This increase was more pronounced in key flavour-associated amino acids. The most substantial increase was observed in glutamic acid, with concentration increasing from 2.7 to 43.0 µmol/L, while aspartic acid, another primary umami contributor, increased from 0.8 to 16.5 µmol/L. Furthermore, sweet-tasting amino acids such as alanine and glycine increased from 10.5 to 53.2 µmol/L and 4.4 to 21.4 µmol/L respectively. The parallel upward trajectory of aromatic precursors like phenylalanine, which increased from 3.5 to 30.3 µmol/L, highlight a comprehensive shift in the FAA profile. This collective increase, most notably in the umami and sweet compounds defined by Yamaguchi et al. (1971), provides a strong biochemical basis for the markedly improved flavour of the fermented product.

Five 5′-nucleotides were targeted, of which four were detected in LMM; 5′-IMP was below the limit of detection (1.7 µmol/L) on all sampling days and is reported as n.d. (Table S3).

Table S3. Change in the 5'-nucleotides content during the fermentation process from Day 0 to Day 10 (µmol/L)

Category	Cytidine-5'-monophosphate	Uridine 5'-monophosphate	Adenosine 5' monophosphate	Guanosine 5'-monophosphate	Inosine 5'-monophosphate
0	3.833 ± 0.49^{e}	9.891 ± 2.68^{e}	1.229 ± 0.08^{e}	6.364 ± 0.77^{e}	n.d.
1	4.698 ± 0.53^{de}	10.693 ± 1.37^{de}	1.364 ± 0.14^{e}	7.419 ± 0.67^{d}	n.d.
2	4.881 ± 0.48^{cd}	10.060 ± 1.02^{e}	1.532 ± 0.48^{de}	7.619 ± 0.59^{cd}	n.d.
3	5.500 ± 1.31^{bcd}	13.001 ± 2.49^{d}	1.913 ± 0.15^{cde}	8.435 ± 0.45^{bc}	n.d.
4	5.409 ± 0.39^{bcd}	17.477 ± 0.76^{c}	2.505 ± 0.45^{bc}	9.037 ± 0.75^{ab}	n.d.
5	5.693 ± 0.84^{bcd}	20.744 ± 1.90^{ab}	4.405 ± 0.60^{a}	9.119 ± 0.14^{ab}	n.d.
6	5.803 ± 0.50^{bc}	23.001 ± 2.49^{a}	4.523 ± 0.57^{a}	9.459 ± 0.18^{a}	n.d.
7	6.426 ± 0.22^{ab}	21.759 ± 2.90^{ab}	3.081 ± 0.19^{b}	9.660 ± 0.13^{a}	n.d.
8	7.177 ± 0.30^{a}	20.060 ± 1.02^{b}	3.066 ± 0.23^{b}	9.704 ± 0.18^{a}	n.d.
9	7.228 ± 0.19^{a}	20.558 ± 1.66^{b}	2.316 ± 0.50^{c}	9.419 ± 0.38^{a}	n.d.
10	7.405 ± 0.17^{a}	20.693 ± 1.37^{ab}	2.147 ± 0.56^{cd}	9.697 ± 0.40^{a}	n.d.
Pr > F(Model)	<0.0001	<0.0001	<0.0001	<0.0001	-
Significant	Yes	Yes	Yes	Yes	
Pr > F(Day)	<0.0001	<0.0001	<0.0001	<0.0001	-

Significant	Yes	Yes	Yes	Yes

The fermentation process significantly altered the 5’-nucleotide profile of the LMM, showing a general trend of increase that paralleled the free amino acids. As detailed in Table S3, the concentrations of four key nucleotides increased significantly ($p < 0.0001$) from Day 0 to Day 10. Specifically, cytidine-5'-monophosphate (5'-CMP) and guanosine 5'-monophosphate (5'-GMP) exhibited steady increases, with their concentrations rising from 3.8 to 7.4 μmol/L and from 6.4 to 9.7 μmol/L, respectively. In contrast, uridine 5'-monophosphate (5'-UMP) and adenosine 5' monophosphate (5'-AMP) showed more dynamic patterns, among which 5′-UMP increased substantially from 9.9 to 20.7 μmol/L, while 5′-AMP increased from 1.2 to a maximum of 4.5 μmol/L on Day 6 before declining to 2.1 μmol/L by Day 10 (values below the LOQ of 4.0 μmol/L are semi-quantitative). Throughout this period, 5′-IMP remained below the limit of detection (1.7 μmol/L) on all sampling days and is reported as not detected (n.d.); no statistical testing was performed for this compound.

This enrichment is critically important for flavour development. 5'-AMP is known to impart a sweet taste and can mask bitter flavours in mushrooms, while 5'-GMP contributes a desirable meaty or brothy umami taste [6, 7]. The synergistic effect of these nucleotides, which may be more effective than monosodium glutamate in enhancing savoury notes [8], signifies that fermentation is a highly effective process for developing the complex, pleasant, and meat-like flavour profile of the LMM.

Five reducing sugars that may be present in LMM were detected including rhamnose, galactose, glucose, glucuronic acid, arabinose, and fucose according to [9]. The results are shown on the table below. Among them, rhamnose, galactose and glucuronic acid could not be detected in LMM, and the three reducing sugars, glucose, arabinose and fucose were detected.

Table S4. Changes in reducing sugar content during fermentation (μmol/L)

Fermentation (Day)	Arabinose	Fucose	Glucose
0	0.975 ± 0.01^{f}	0.903 ± 0.14^{b}	660.566 ± 40.02^{d}
1	2.266 ± 0.41^{e}	1.367 ± 0.33^{a}	905.197 ± 101.89^{a}
2	3.482 ± 0.67^{cd}	0.976 ± 0.22^{b}	831.110 ± 94.50^{abc}
3	2.897 ± 0.38^{de}	1.092 ± 0.06^{ab}	755.286 ± 37.45^{cd}
4	3.403 ± 0.32^{cd}	0.837 ± 0.10^{b}	766.040 ± 76.91^{cd}
5	3.698 ± 0.43^{bc}	0.904 ± 0.20^{b}	808.650 ± 92.26^{abc}
6	3.618 ± 0.13^{c}	0.924 ± 0.14^{b}	765.569 ± 24.28^{cd}
7	4.396 ± 0.87^{ab}	1.028 ± 0.12^{b}	823.635 ± 159.29^{abc}
8	4.693 ± 0.68^{a}	0.980 ± 0.16^{b}	885.701 ± 55.53^{ab}
9	4.567 ± 0.17^{a}	0.881 ± 0.09^{b}	784.228 ± 33.83^{bc}
10	4.804 ± 0.31^{a}	0.830 ± 0.19^{b}	844.045 ± 59.07^{abc}
Pr > F(Model)	<0.0001	0.036	0.012
Significant	Yes	Yes	Yes
Pr > F(Day)	<0.0001	0.038	0.022
Significant	Yes	Yes	Yes

The fermentation process differentially influenced the content of individual reducing sugars in the LMM, as detailed in Table S4. Arabinose exhibited a significant and sustained increasing trend ($p < 0.0001$), with its concentration rising from 0.98 μmol/L on Day 0 to 4.80 μmol/L on Day 10. In contrast, glucose displayed a more dynamic profile, wherein its content surged from 660.57 μmol/L to a peak of 905.20 μmol/L on Day 1, before gradually decreasing and stabilising around 844.05 μmol/L by the end of fermentation. Meanwhile, fucose levels remained relatively stable throughout the process, showing no consistent trend despite statistical significance.

The initial sharp increase in glucose can be attributed to the rapid hydrolysis of polysaccharides like starch by *Lactobacillus* [10]. The subsequent decline likely reflects accelerated microbial consumption of glucose as a primary carbon source for energy and growth. In the later stages, the stabilisation of glucose levels may be due to depleted polysaccharide reserves and an inhibitory low pH environment that curtails further microbial activity [11]. The continuous accumulation of arabinose, even as glucose is consumed, suggests it may be a structural or metabolic by-product that is not preferentially utilised by the microbiota under these conditions. Similarly, the stability

of fucose implies its poor bioavailability or complex metabolic pathways, preventing its significant participation in the fermentation [12].

The fermentation process significantly modulated the antioxidant profile of LMM, as evidenced by the data for TPC and antioxidant activities presented in Table S5.

Table S5. Change in TPC and antioxidant activities during fermentation (TPC: mg gallic acid equivalent/g; CUPRAC and FRAP: mg ascorbic acid equivalent/g)

Category	CUPRAC	FRAP	TPC
0	2.165 ± 0.01^{d}	0.682 ± 0.03^{cd}	1.773 ± 0.02^{h}
1	2.220 ± 0.06^{c}	0.567 ± 0.03^{f}	1.480 ± 0.01^{j}
2	2.058 ± 0.06^{f}	0.419 ± 0.03^{h}	1.629 ± 0.01^{i}
3	2.142 ± 0.01^{d}	0.497 ± 0.04^{g}	1.926 ± 0.01^{f}
4	2.266 ± 0.03^{c}	0.604 ± 0.06^{ef}	1.876 ± 0.01^{g}
5	2.342 ± 0.03^{b}	0.662 ± 0.08^{de}	1.752 ± 0.01^{h}
6	2.373 ± 0.02^{ab}	0.736 ± 0.03^{bc}	1.960 ± 0.01^{e}
7	2.416 ± 0.01^{a}	0.826 ± 0.01^{a}	2.123 ± 0.01^{d}
8	2.348 ± 0.03^{b}	0.715 ± 0.04^{bcd}	2.250 ± 0.01^{c}
9	2.134 ± 0.01^{de}	0.818 ± 0.03^{a}	2.379 ± 0.01^{a}
10	2.082 ± 0.01^{ef}	0.744 ± 0.03^{b}	2.352 ± 0.01^{b}
Pr > F(Model)	<0.0001	<0.0001	<0.0001
Significant	Yes	Yes	Yes
Pr > F(Day)	<0.0001	<0.0001	<0.0001
Significant	Yes	Yes	Yes

The fermentation process significantly changed the antioxidant profile of LMM, as evidenced by the data for TPC and antioxidant activities presented in Table S5. All three metrics showed statistically significant changes over time ($p < 0.0001$), albeit with distinct temporal patterns. The TPC demonstrated a clear and substantial increasing trend, rising from 1.77 mg GAE/g on Day 0 to a peak of 2.38 mg GAE/g on Day 9. This was accompanied by a similar, though more variable, upward trajectory in the FRAP activity, which increased from 0.68 to a peak of 0.82 mg AAE/g on Day 9 (0.744 mg AAE/g on Day 10). In contrast, the CUPRAC activity exhibited a more complex and non-linear profile, with values fluctuating throughout the fermentation and showing no consistent net increase by Day 10.

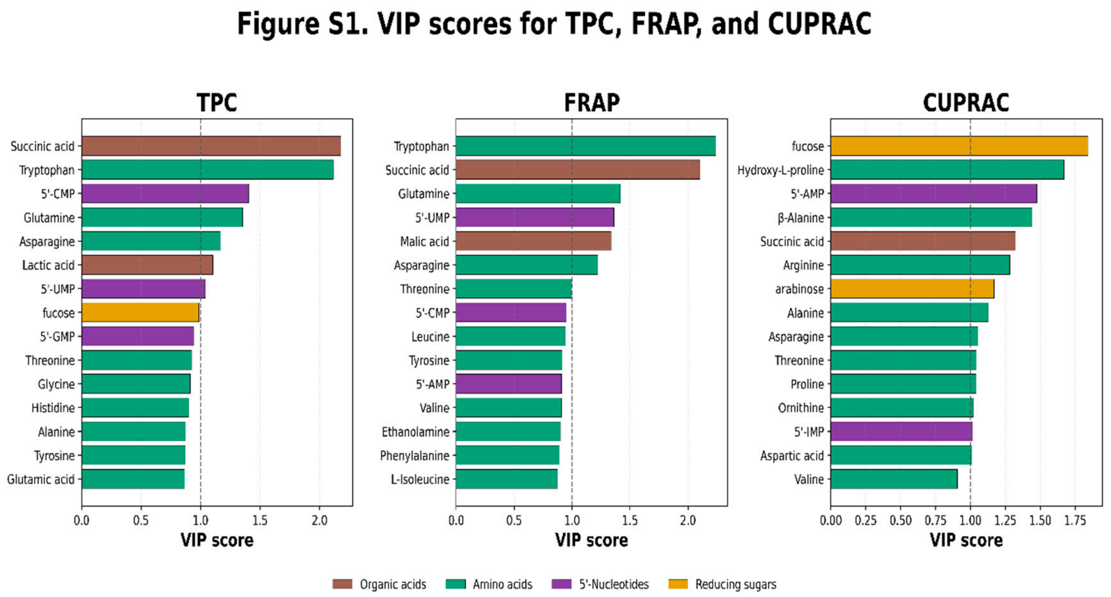

Figure S1. Variable Importance in Projection (VIP) scores for individual PLSR models of Total Phenolic Content (TPC), Ferric Reducing Antioxidant Power (FRAP), and Cupric Reducing Antioxidant Capacity (CUPRAC)

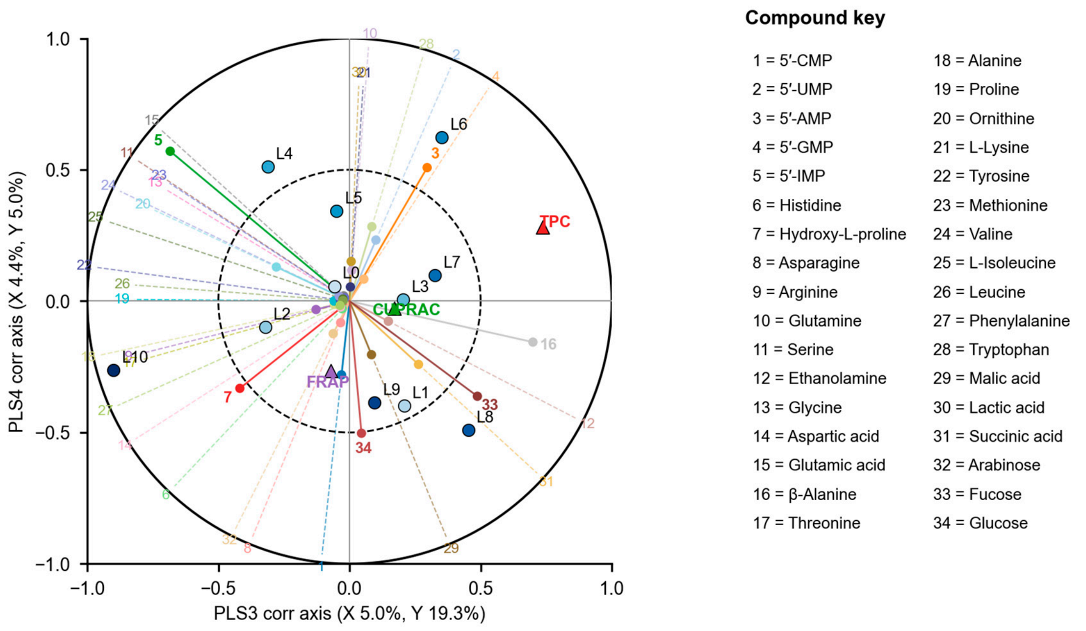

Figure S2. PLS3–PLS4 correlation biplot of the PLSR model applied to the day-averaged non-volatile matrix (n = 3 per day). Numbered points are compound loadings (1–34, see key); labelled triangles are TPC, CUPRAC and FRAP; sample scores are scaled for display within the unit circle. PLS3 and PLS4 together explained an additional 9.4% of X and 24.3% of Y variance.

Note: The vertical dashed line at a VIP score of 1.0 indicates the threshold for importance in the model. Compounds with VIP values > 1.0 are considered key drivers

of the respective antioxidant response. Colour coding identifies the chemical class of each metabolite: organic acids (brown), amino acids (green), 5′-nucleotides (purple), and reducing sugars (yellow).

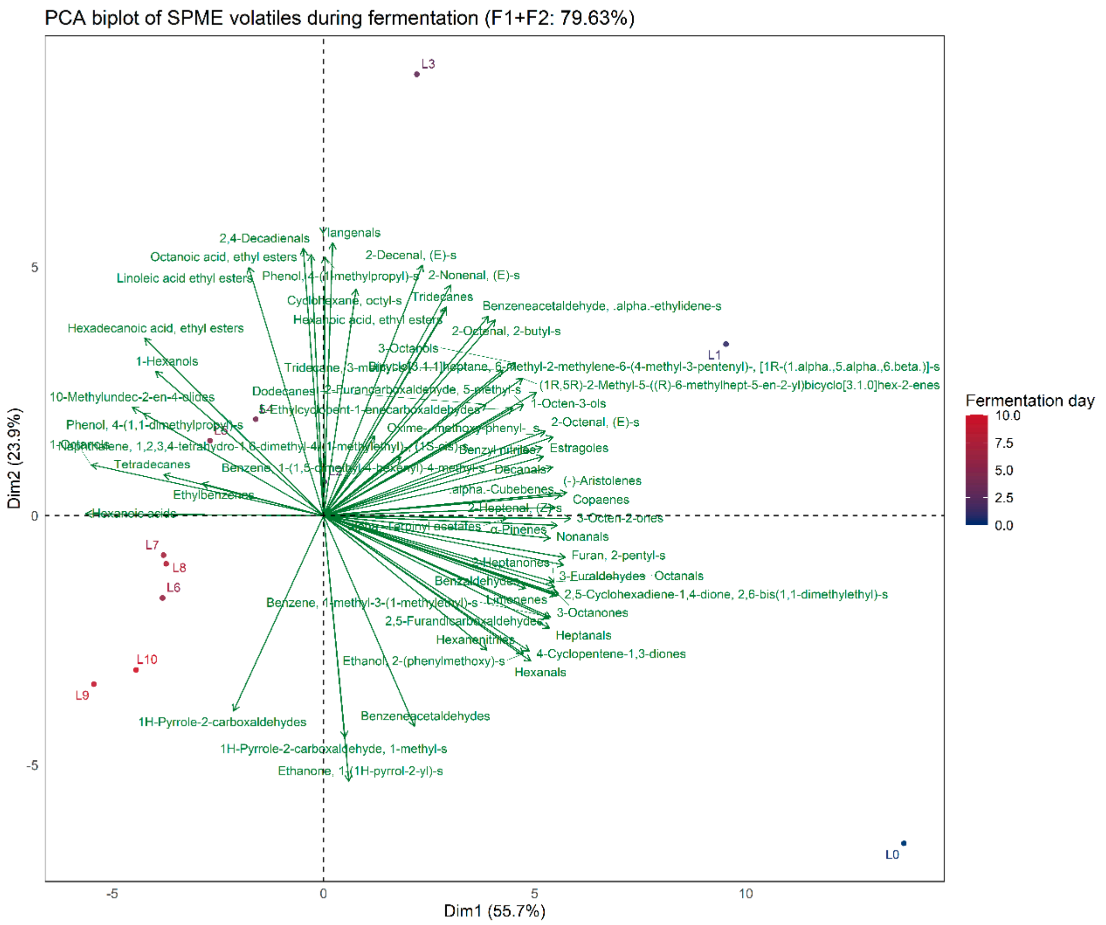

Figure S3. Principal Component Analysis (PCA) biplot of all tentatively identified volatile compounds (n = 62) and LMM samples throughout the fermentation process. All volatile compounds identified by NIST14 library matching are labelled.

Table S6. Volatile compounds tentatively identified in fermented Lingzhi mushroom mycelium (LMM). Values are relative abundances expressed as a percentage of the total peak area (mean of n = 3 biological replicates; —, not detected).

Compound	Class	D0	D1	D2	D3	D4	D5	D6	D7	D8	D9	D10
Hexanal	Aldehyde	2.16	0.76	0.34	0.32	0.32	0.31	0.58	0.56	0.59	0.58	0.56
3-Furaldehyde	Furan	41.51	38.97	28.57	20.38	15.95	17.27	14.90	13.53	15.19	16.07	18.68
Ethylbenzene	Hydrocarbon	0.68	0.91	1.30	1.40	2.21	1.74	2.12	2.12	2.04	2.17	1.92
1-Hexanol	Alcohol	0.15	0.22	2.84	4.51	4.94	4.19	5.17	4.89	4.41	3.32	2.25
Hexanenitrile	Nitrile	0.11	0.04	0.03	0.05	0.12	0.08	0.10	0.09	0.11	0.06	0.05
4-Cyclopentene-1,3-dione	Ketone	0.16	0.05	0.03	0.03	0.05	0.03	0.04	0.05	0.06	0.02	0.03
2-Heptanone	Ketone	0.30	0.14	0.13	0.17	0.10	0.09	0.09	0.07	0.07	0.06	0.06
Heptanal	Aldehyde	0.74	0.49	0.15	0.13	0.12	0.13	0.23	0.20	0.21	0.22	0.22
Oxime-, methoxy-phenyl-_	Other N-compound	3.74	3.39	3.09	4.76	2.97	5.15	1.98	2.51	1.44	0.19	2.38
α-Pinene	Terpene/terpenoid	0.14	0.11	0.14	0.14	0.13	0.14	0.06	0.10	0.16	0.14	0.09
2-Heptenal, (Z)-	Aldehyde	0.93	1.25	0.79	0.89	0.70	0.53	1.01	0.72	0.64	0.62	0.63
2-Furancarboxaldehyde, 5-methyl-	Furan	2.64	3.79	5.10	4.61	5.34	4.84	5.07	4.41	4.71	4.10	4.09
Benzaldehyde	Aldehyde	11.48	12.86	11.68	10.95	16.01	14.21	11.97	17.04	14.84	19.07	16.15
1-Octen-3-ol	Alcohol	7.74	9.02	11.52	13.59	12.47	11.48	11.37	9.72	9.19	7.45	5.44
3-Octanone	Ketone	0.35	0.26	0.16	0.21	0.15	0.16	0.22	0.19	0.17	0.19	0.19
Furan, 2-pentyl-	Furan	2.04	1.77	1.60	2.01	1.85	1.90	2.04	1.64	1.95	1.81	1.97
Hexanoic acid	Organic acid	3.02	3.44	7.56	8.58	10.44	11.05	13.08	12.57	13.08	12.72	12.37
3-Octanol	Alcohol	0.46	0.55	0.76	0.91	0.87	0.85	0.90	0.80	0.71	0.63	0.50
Hexanoic acid, ethyl ester	Ester	0.12	0.52	0.58	0.62	0.44	0.34	0.28	0.56	0.12	0.08	0.06
Octanal	Aldehyde	0.46	0.48	0.14	0.12	0.10	0.10	0.19	0.14	0.15	0.17	0.16
1H-Pyrrole-2-carboxaldehyde	Aldehyde	0.06	0.07	0.13	0.06	0.13	0.13	0.19	0.17	0.17	0.16	0.17
Benzene, 1-methyl-3-(1-methylethyl)-	Hydrocarbon	0.94	0.43	0.23	0.27	0.21	0.20	0.14	0.14	0.14	0.17	0.14
Limonene	Terpene/terpenoid	2.88	1.81	1.34	1.65	1.27	1.31	0.80	0.98	1.41	1.68	1.09
5-Ethylcyclopent-1-enecarboxaldehyde	Aldehyde	0.13	0.40	0.22	0.23	0.12	0.11	0.08	0.05	0.07	0.06	0.05
3-Octen-2-one	Ketone	0.50	0.43	0.27	0.38	0.21	0.16	0.12	0.10	0.09	0.07	0.06

Benzeneacetaldehyde	Aldehyde	6.64	4.43	4.59	4.50	5.04	5.61	7.18	8.15	9.54	11.73	13.77
2-Octenal, (E)-	Aldehyde	0.72	1.22	0.87	1.00	0.73	0.65	0.66	0.48	0.45	0.42	0.35
Ethanone, 1-(1H-pyrrol-2-yl)-	Other	0.56	0.47	0.89	0.37	0.76	0.94	1.34	1.22	1.17	1.11	1.19
1-Octanol	Alcohol	0.07	0.10	0.30	0.38	0.46	0.41	0.53	0.55	0.57	0.54	0.49
2,5-Furandicarboxaldehyde	Furan	1.48	0.92	1.19	0.43	0.43	0.52	0.55	0.48	0.42	0.44	0.71
Nonanal	Aldehyde	1.27	1.62	1.08	0.86	0.63	0.78	0.64	0.71	0.68	0.70	0.67
1H-Pyrrole-2-carboxaldehyde, 1-methyl-	Aldehyde	0.08	0.09	0.17	0.06	0.13	0.15	0.21	0.20	0.18	0.19	0.20
Benzyl nitrile	Nitrile	0.10	0.09	0.13	0.16	0.16	0.16	0.15	0.15	0.14	0.14	0.12
2-Nonenal, (E)-	Aldehyde	0.24	0.41	0.55	0.63	0.60	0.63	0.62	0.54	0.59	0.52	0.47
Estragole	Terpene/terpenoid	0.21	0.18	0.20	0.33	0.23	0.20	0.17	0.15	0.17	0.13	0.12
Octanoic acid, ethyl ester	Ester	0.04	0.31	0.52	0.60	0.58	0.52	0.51	0.57	0.36	0.27	0.20
Dodecane	Hydrocarbon	1.22	1.44	1.84	2.50	2.32	2.13	3.00	2.67	2.65	2.21	2.65
Decanal	Aldehyde	0.11	0.15	0.15	0.17	0.15	0.16	0.17	0.18	0.18	0.16	0.17
Ethanol, 2-(phenylmethoxy)-	Other	0.14	0.02	0.01	0.01	0.01	0.00	0.00	0.00	0.00	0.00	0.00
2-Decenal, (E)-	Aldehyde	0.10	0.23	0.26	0.35	0.31	0.31	0.31	0.27	0.27	0.25	0.22
Benzeneacetaldehyde, .alpha.-ethylidene-	Aldehyde	0.21	0.28	0.39	0.47	0.47	0.46	0.42	0.42	0.44	0.41	0.36
Tridecane	Hydrocarbon	1.31	1.94	2.48	3.05	2.97	2.93	3.73	3.20	3.28	2.72	3.09
Phenol, 4-(1-methylpropyl)-	Phenolic	0.07	0.94	1.20	1.37	1.29	1.26	1.18	1.02	1.01	0.86	0.71
2,4-Decadienal	Aldehyde	0.12	0.87	1.38	1.59	1.50	1.48	1.21	1.10	1.08	1.07	0.85
(-)-Aristolene	Terpene/terpenoid	0.04	0.04	0.04	0.05	0.05	0.04	0.06	0.05	0.05	0.04	0.04
.alpha.-Terpinyl acetate	Ester	0.13	0.09	0.12	0.20	0.13	0.13	0.14	0.16	0.22	0.12	0.17
2-Octenal, 2-butyl-	Aldehyde	0.05	0.06	0.08	0.10	0.10	0.10	0.11	0.10	0.11	0.09	0.09
Tridecane, 3-methyl-	Hydrocarbon	0.13	0.15	0.21	0.26	0.26	0.26	0.31	0.30	0.31	0.25	0.27
.alpha.-Cubebene	Terpene/terpenoid	0.21	0.19	0.21	0.30	0.28	0.24	0.30	0.28	0.31	0.24	0.24
Copaene	Terpene/terpenoid	0.21	0.19	0.21	0.30	0.28	0.24	0.30	0.28	0.31	0.24	0.24
Phenol, 4-(1,1-dimethylpropyl)-	Phenolic	0.37	0.44	0.72	0.81	0.89	0.96	1.08	1.06	1.11	1.01	1.01
Tetradecane	Hydrocarbon	0.21	0.27	0.39	0.52	0.53	0.57	0.69	0.67	0.72	0.65	0.71

(1R,5R)-2-Methyl-5-((R)-6-methylhept-5-en-2-yl)bicyclo[3.1.0]hex-2-ene	Terpene/terpenoid	0.04	0.05	0.06	0.08	0.08	0.08	0.09	0.08	0.09	0.07	0.08
Bicyclo[3.1.1]heptane, 6-methyl-2-methylene-6-(4-methyl-3-pentenyl)-, [1R-(1.alpha.,5.alpha.,6.beta.)]-	Terpene/terpenoid	0.04	0.05	0.06	0.08	0.08	0.08	0.09	0.08	0.09	0.07	0.08
Cyclohexane, octyl-	Hydrocarbon	0.06	0.09	0.13	0.14	0.15	0.16	0.17	0.18	0.18	0.15	0.16
2,5-Cyclohexadiene-1,4-dione, 2,6-bis(1,1-dimethylethyl)-	Ketone	0.07	0.07	0.09	0.09	0.10	0.09	0.12	0.10	0.12	0.11	0.10
Benzene, 1-(1,5-dimethyl-4-hexenyl)-4-methyl-	Hydrocarbon	0.08	0.08	0.10	0.17	0.14	0.14	0.16	0.17	0.22	0.14	0.19
Naphthalene, 1,2,3,4-tetrahydro-1,6-dimethyl-4-(1-methylethyl)-, (1S-cis)-	Hydrocarbon	0.10	0.11	0.17	0.19	0.20	0.21	0.24	0.24	0.27	0.20	0.24
10-Methylundec-2-en-4-olide	Lactone	0.07	0.11	0.19	0.31	0.29	0.31	0.33	0.35	0.38	0.39	0.33
Ylangenal	Terpene/terpenoid	0.06	0.08	0.12	0.16	0.15	0.16	0.16	0.15	0.17	0.15	0.14
Hexadecanoic acid, ethyl ester	Ester	0.01	0.08	0.19	0.35	0.28	0.33	0.31	0.30	0.38	0.33	0.24
Linoleic acid ethyl ester	Ester	0.00	0.02	0.04	0.08	0.04	0.05	0.05	0.04	0.06	0.05	0.03

Reference

1. Özcelik, S., E. Kuley, and F. Özogul, *Formation of lactic, acetic, succinic, propionic, formic and butyric acid by lactic acid bacteria.* Lwt, 2016. **73**: p. 536-542.

2. Xu, Y., Y. Cui, X. Wang, F. Yue, Y. Shan, B. Liu, Y. Zhou, Y. Yi, and X. Lü, *Purification, characterization and bioactivity of exopolysaccharides produced by Lactobacillus plantarum KX041.* International journal of biological macromolecules, 2019. **128**: p. 480-492.

3. Magdziak, Z., M. Siwulski, and M. Mleczek, *Characteristics of organic acid profiles in 16 species of wild growing edible mushrooms.* Journal of Environmental Science and Health, Part B, 2017. **52**(10): p. 784-789.

4. Fuke, S. and S. Konosu, *Taste-active components in some foods: a review of Japanese research.* Physiology & Behavior, 1991. **49**(5): p. 863-868.

5. Bu, X., X. Xue, J. Cheng, and S. Liu, *Evaluation on malolactic fermentation potential of wine Lactobacillus plantarum.* Scientia Agricultura Sinica, 2017. **50**: p. 959-968.

6. Leksrisompong, P., P. Gerard, K. Lopetcharat, and M. Drake, *Bitter taste inhibiting agents for whey protein hydrolysate and whey protein hydrolysate beverages.* Journal of food science, 2012. **77**(8): p. S282-S287.

7. Chen, Z., X. Fang, W. Wu, H. Chen, Y. Han, H. Yang, and H. Gao, *Effects of fermentation with Lactiplantibacillus plantarum GDM1.191 on the umami compounds in shiitake mushrooms (Lentinus edodes).* Food Chemistry, 2021. **364**: p. 130398.

8. Yue, J., Y. Zhang, Y. Jin, Y. Deng, and Y. Zhao, *Impact of high hydrostatic pressure on non-volatile and volatile compounds of squid muscles.* Food Chemistry, 2016. **194**: p. 12-19.

9. Wang, Q., F. Wang, Z. Xu, and Z. Ding, *Bioactive mushroom polysaccharides: a review on monosaccharide composition, biosynthesis and regulation.* Molecules, 2017. **22**(6): p. 955.

10. Bing, H., Z. Xuzhen, L.I. Amin, Y. Ruilian, and J. Heti, *Effect of Lactic Acid Bacterial Fermentation on the Quality of Kohlrabi.* Food Science, 2016.

11. Hong-Xia, L., Z. Xing-Yuan, W. Jian, L. Shao-Hua, and J. Hong-Liang, *Study on the Technology of Fermenting Compound Vegetables with Lactic Acid Bacteria.* China Fruit & Vegetable, 2018.

12. Becerra, J.E., M.J. Yebra, and V. Monedero, *An L-fucose operon in the probiotic Lactobacillus rhamnosus GG is involved in adaptation to gastrointestinal conditions.* Applied and Environmental Microbiology, 2015. **81**(11): p. 3880-3888.
